# Sialylated *N*-glycan profile during acute and chronic infections with *Toxoplasma gondii* in mice

**DOI:** 10.1038/s41598-020-60681-4

**Published:** 2020-03-02

**Authors:** Ibrahim Farag Rehan, Motamed Elsayed Mahmoud, Doaa Salman, Asmaa Elnagar, Saleh Salman, Mohammed Youssef, Amer Ragheb Abdel Aziz, Eman Kamal Bazh, Abd El-Latif Hesham

**Affiliations:** 1 Department of Husbandry and Development of Animal Wealth, Faculty of Veterinary Medicine, Menofia University, Shebin Alkom, Menofia 32511 Egypt; 2 Department of Animal Behavior and Husbandry (management, genetics, and breeding), Faculty of Veterinary Medicine, Sohag University, Sohag, 82524 Egypt; 3 Department of Animal Medicine, Faculty of Veterinary Medicine, Sohag University, Sohag, 82524 Egypt; 4 Department of Biochemistry, Faculty of Veterinary Medicine, South Valley University, Qena, 83523 Egypt; 5 Department of Animal Sciences, Colorado State University, Fort Collins, 80523 Colorado USA; 6 Department of Animal Production, Faculty of Agriculture, Assiut University, Assiut, 71111 Egypt; 7 Department of Animal Physiology, Faculty of Veterinary Medicine, South Valley University, Qena, 83523 Egypt; 8 Department of Parasitology, Faculty of Veterinary Medicine, Sohag University, Sohag, 82524 Egypt; 9 Department of Parasitology, Faculty of Veterinary Medicine, Menofia University, Shebin Alkom, Menofia 32511 Egypt; 10 Department of Genetics, Faculty of Agriculture, Beni-Suef University, Beni-Suef, 62511 Egypt

**Keywords:** Glycosylation, Drug discovery, Glycosylation, Diagnostic markers

## Abstract

*Toxoplasma gondii* is associated with physiological and psychiatric perturbations. The immune response is interrelated to the progress of anhedonia and despair symptoms of *T. gondii*-infected subjects. We recently reported that serum *N*-glycans were altered in mice displayed depressive-like behaviors. However, a novel biomarker that correlated to *T. gondii* infection and associated behaviors is demanded. Glycomics has been used to find affected glycoproteins during depression. The objective of this study is to investigate serum *N*-glycomics changes during infection with *T. gondii* in BALB/c mice, immunocompetent, or in severe combined immunodeficient mice, and after treatment with an immunostimulant; 1-methyl tryptophan. Glycans were examined through glycoblotting-protocol then investigated by MALDI-TOF/MS. Both depressive and sickness-related behaviors were significantly abundant (*P* ≤ 0.001 each), during acute *T. gondii* in immunocompetent mice, compared to controls. Only sickness symptoms were evident in immunodeficient mice infected with *T. gondii*, as associated with high expression level (*P* ≤ 0.001) of Peak # 15 (2 × Neu5Gc) compared to controls. The alteration of sialylated *N*-glycan expressions is important to detect the immune status of animals/humans against *T. gondii*. Moreover, 1-methyl tryptophan reduced depressive-like behavior (*P* ≤ 0.001) compared to controls. Therefore, sialylated *N*-glycan (Neu5Ac/Neu5Gc-terminal) is targeted to be used as a novel biomarker of sickness/depressive-like behaviors.

## Introduction

*Toxoplasma gondii* affects all warm-blooded animals and infects almost one-third of all humans. *T. gondii* infection is a parasitic disease prevalent worldwide and involved in many psychiatric disorders^1^. Moreover; depression is considered as one of the major psychological disorders and the global root of disability in the 21^st^ century^2^. It is defined as a mood disorder due to the reduction of interest, happiness, learning abilities, sleep, appetite, and energy^3^. Several studies had shown that seroprevalence of *T. gondii* in neuropsychiatric defects were variable like schizophrenia, bipolar mood disorder^4^ and self-directed violence^5^. Challenge with *T. gondii* revealed two characteristic forms: immune-stimulating tachyzoites and immune-encrypted bradyzoites. Recent studies had demonstrated that the brain is an immune-privileged region of residence of bradyzoite cysts^6^. Hence, the pathogenesis of infection depends mainly upon host immunity activated macrophages and lymphocytes immediately primed to kill intracellular tachyzoites^7^. However, under lowered immunity, the propagation of tachyzoites can be quite high as shown in immune-competent individuals^8^, which may result in fatal toxoplasmic encephalitis^9^. *T. gondii* reactivation in chronic infection was reported to lead to depression-related behaviors in BALB/c mice^6^. Because of contradictory information on the mechanisms that link *T. gondii* infection to bipolar depressive disorder^4,5,10^ reveals the need to investigate the biological correlates of sickness as well as depressive-like behaviors in *T. gondii* infection.

Glycomic has been utilized to find the altered glycoproteins and their pathways in depression, which has provided a clear understanding of depression molecular mechanisms and its potential role in therapy. Moreover, energy metabolism and synaptic pathways have often been linked to depression^9^ and with the antidepressant treatment response^11,12^. The glycosylation process is considered the enzymatic activities which generate sugar chains associated with proteins as well as lipids. *N*-glycans allocated on proteins are existed as oligosaccharides and mainly attached to proteins at its asparagine residues via the *N*-glycosidic-bond, suggesting the importance of glycomics in multi-biological/physiological properties^13^. These functions include protein localization, protein folding, cell adhesion, and cell-cell interaction^14^. A recent study has shown that several glycosylation enzymes (e.g., *sialyltransferase*) have strongly interacted with the pathobiology of psychiatric disorders^1^. It is well-established that total plasma *sialyltransferase* levels plus cortisol affect HPA axis function^15^ and alter the gene expression^16^. Importantly, *N*-glycosylation is the first line of defense to control the function of immune system^13^. In addition, *N*-glycosylation has a potential effect on the other neuropsychiatric disorders like post-traumatic stress disorder^16^, schizophrenia^17^, and stress-induced depressive behaviors in mice^18^. Moreover, we recorded the inability of the homeostatic immune system of BALAB/c mice to cope the chronic stressors. Recently, the involvement of *T. gondii* in the assembly of novel glycans within infected fibroblasts was demonstrated^19^. Here, we reported that the ratio of serum *N*-glycans terminated with Neu5Gc/Neu5Ac evidenced the heat stress and elevated the milk production curve as well as maternity responses of Holstein dairy cows^20^. Although earlier prognosis is crucial to therapy efficiency, the difference between the onset of sickness duration and depressive-like signs in *T. gondii* is still poorly understood and needs more clarifications.

Therefore, the current study investigates the changes of serum glycans as biological correlates for acute and chronic phases of *T. gondii* infection. Glycoblotting is widely considered an exact way to purify glycans of sera and cell cultures samples^20^. It is an applicable technique, especially in large-scale analysis for glycomics in serum glycoproteins, since the analytic-plate can involve 96 samples in time of analysis. To perform this designated bead-platform, a few samples volume (<100 *μ*L) was required for the “SweetBlot” along with matrix-assisted laser desorption ionization-time of flight/mass spectrometry (MALDI-TOF/MS)^21^. Interestingly, glycoform analysis was completed within 12–18 hours. Furthermore; the MS analysis is also regarded for its speediness and accuracy in quantifying glycans and its role in the discovery of several medical diseases and stress biomarkers in human and animals^20^. Here, we employed glycoblotting strategy then together with MALDI-TOF/MS to find the estimated structures of serum *N*-glycans and their expression levels/peaks to clarify the glycomics of experimental groups and control in BALB/c mice, in correlation to mouse’s behavioral results.

BALB/c mice are an ideal model of immunity challenge to exhibit behaviors characteristic for sickness as well as depressive-like behaviors since these mice are relatively resistant to *T. gondii* and can develop chronic latent infection^14^. Importantly, treatment with 1-methyl tryptophan (1-MT) reduced sickness-and depressive-like behaviors of *T. gondii*, besides lowered the parasite burden in brains of mice, which indicate the role of *T. gondii* immune response in promoting depressive behavior^1^. Therefore, BALB/c mice are predicted to show more depressive-like behaviors than sickness signs. We chose different strategies to assess sickness such as clinical scoring, locomotor abilities, and depression states as anhedonic-and despair-related behaviors in BALB/c mice. A previous study has shown that sucrose preference and/or motility in forced-swim was reduced through the acute stage of *T. gondii* infection *T. gondii* in BALB/c mice compared to the controls^22^. Also, the same treatment with 1-MT was used to block indoleamine 2, 3-dioxygenase (IDO) functions^23^. Thus, in this study, we examined the biological importance of serum *N*-glycan of mice infected with acute/chronic *T. gondii* on behavioral records. We also analyzed the glycobiological correlates with depressive-like behavior and investigated whether serum *N*-glycome biosignatures are related to severe depressive signs and 1-MT treatment responses or not. We used glycoblotting accompanied by MALDI-TOF/MS^24^, in the study to discuss the changes in the biosynthetic pathway of serum *N*-glycome profiles of chronic-, acute-*T. gondii* infection & treatment, and immunodeficiency syndrome in BALB/c mice *in vivo*. Clinically, 1-_DL_-MT could be used as a kind of adjuvant to prepare vaccine and an add-on immune-stimulant drug for cancer-patients^25^. As far as IDO triggers intracellular tryptophan depletion, it prevents the multiplications of T-cells which permit cancer tissues to evade from the body immune system^26^. Hence, IDO inhibitor 1-_DL_-MT antagonizes with immuno-suppressive influences of IDO drug^27^. Although chronic therapy, either dealing with 1-_L_-MT or 1-_DL_-MT shows controversial anti-parasitic activities, the sub-chronic treatment with 1-_DL_-MT shows some anti-toxoplasma activity^28,29^. Moreover, IDO-deficient mice or with IDO-blocked through MT drug exhibit a reduction of mRNA expression of *T. gondii* in the lungs^29^. This helps us understand the biological significance of the glycome findings *in vitro* to the animal behavioral data *in vivo*. So, the study was designed to test the significance of glycomics in both immunocompetent and immunodeficient states of BALB/c mice sera and to investigate their *N*-glycan profiles. Our results have clearly indicated the biological significance of sialylated *N*-glycans and its validity when used as an effective biomarker for depression and anxiety during *T. gondii* infection. Consequently, sialylated *N*-glycan provides a better understanding of modulating behavioral changes associated with *T. gondii* infection.

## Results

### Behaviors displayed in *T. gondii* infected immunocompetent BALB/c mice

**Figure 1:**
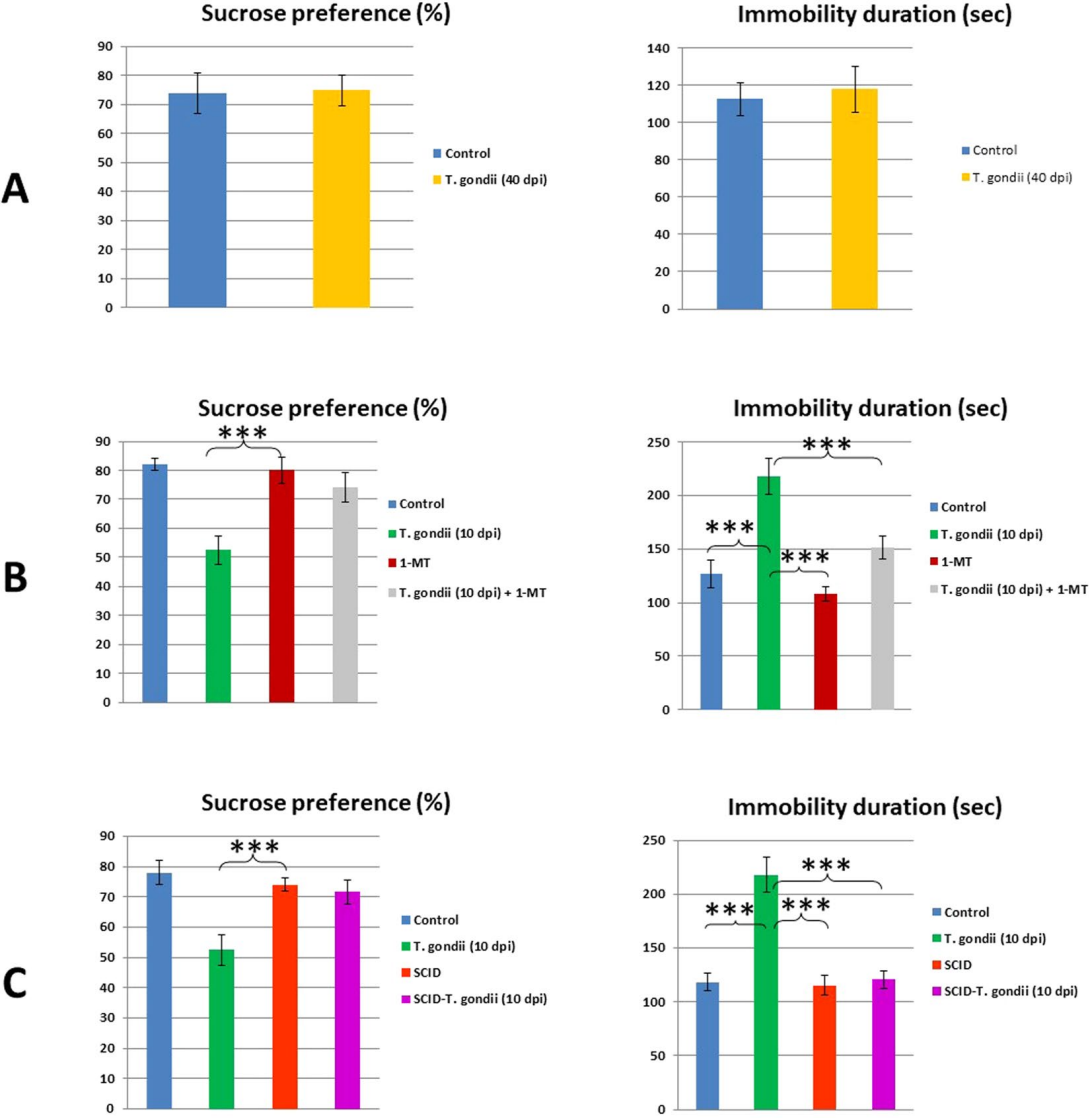
Developed anhedonic-and despair-like behaviors after *T. gondii* infection at 10 day-post infection (10 dpi) in immunocompetent but not in SCID mice. Reduced sucrose preference, as proportion of the complete fluid consumption over 24 hours, immobility duration was measured in *T. gondii*-infected and control mice (40 dpi) **(A)**, after treatment with 1-MT at 10 dpi **(B)**, and in SCID mice at 10 dpi **(C)**. Data represent as mean ± SD.

**Figure 2:**
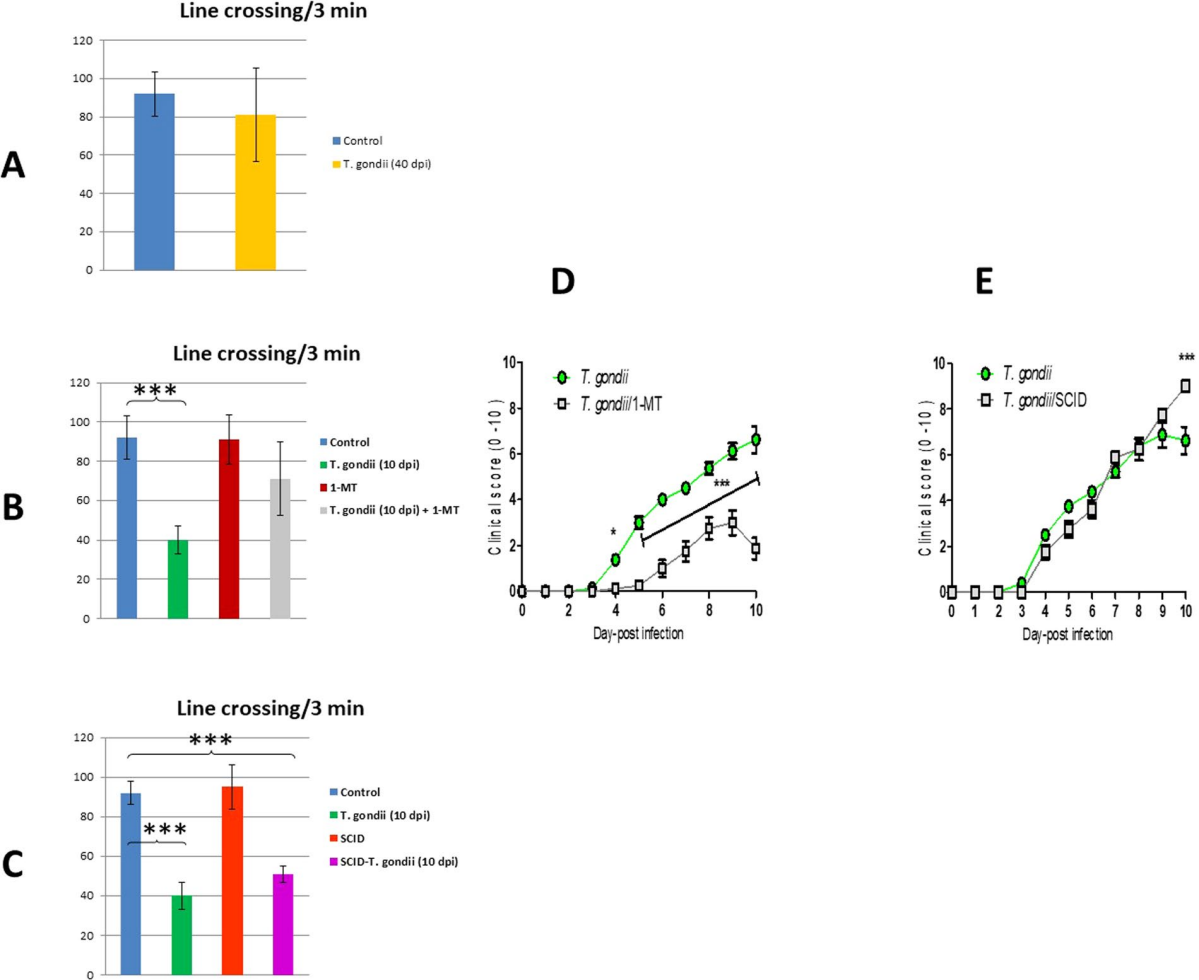
Sickness-like behaviors were examined through line crossing/3 min of *T. gondii*-infected and control mice (40 dpi) **(A)**, after treatment with 1-MT at 10 dpi **(B)**, and in SCID mice at 10 dpi **(C)**, also through clinical scoring from day-0 to day-10 in groups of mice infected with *T. gondii* and then the other handled with 1-MT **(D)**, and in groups of BALB/c infected with *T. gondii* and the other infected SCID mice **(E)**, and 1-MT, 1-methyl tryptophan.

The experiment-I results showed no significant differences in sucrose consumption, immobility in FST, and locomotor activity (illustrated in Figs. 1A, 2A) from mice at 30 days post-infection (dpi) with *T. gondii* and control mice. Also, the customized ethogram for *T. gondii* did not distinguish between chronically-infected BALB/c mice at 40 dpi and controls. Thus, these results refer to neither depressive nor sickness-like behaviors were exhibited in chronically infected immunocompetent BALB/c mice with *T. gondii*.

In experiment-II, using 2 × 2 factorial experimental design, depressive-like behaviors were observed in mice at 10 dpi as exhibited by significantly increased immobility in the FST and reduced locomotor activity (Figs. 1B, 2B). Moreover, there is an increase in clinical score (see Fig. 2D) of sickness and significantly decreased sucrose consumption, which is the hallmark of anhedonic-like behaviors. *T. gondii*-infected and 1-MT-treated mice vs control groups, 1-MT treatment clearly reduced the clinical score, sucrose preference, and immobility in FST compared to mice at 10 dpi. To sum, depressive-and sickness-like behaviors were exhibited during acute *T. gondii* infection and 1-MT treatment attenuated these behaviors in BALB/c mice.

### Sickness-but, not depressive-like behaviors developed in genetically immunodeficient mice

In experiment-III, sucrose consumption and immobility time in FST of infected severely compromised immunodeficient (SCID) mice were relatively the same after exposure to *T. gondii* infection comparable to BALB/c mice (Fig. 1C). Our results also showed no differences among *T. gondii* infected- SCID and control mice in both anhedonic-and despair-related behaviors. It may indicate that infection of *T. gondii* did not induce despair-like behavior in SCID mice. However, the infection reduced the locomotion significantly and elevated the clinical score in such mice (Fig. 2C,E). The *T. gondii*- infected SCID mice may die after day-10.

### MALDI-TOF/MS-analysis of *N*-glycans (experiment I-III)

**Figure 3:**
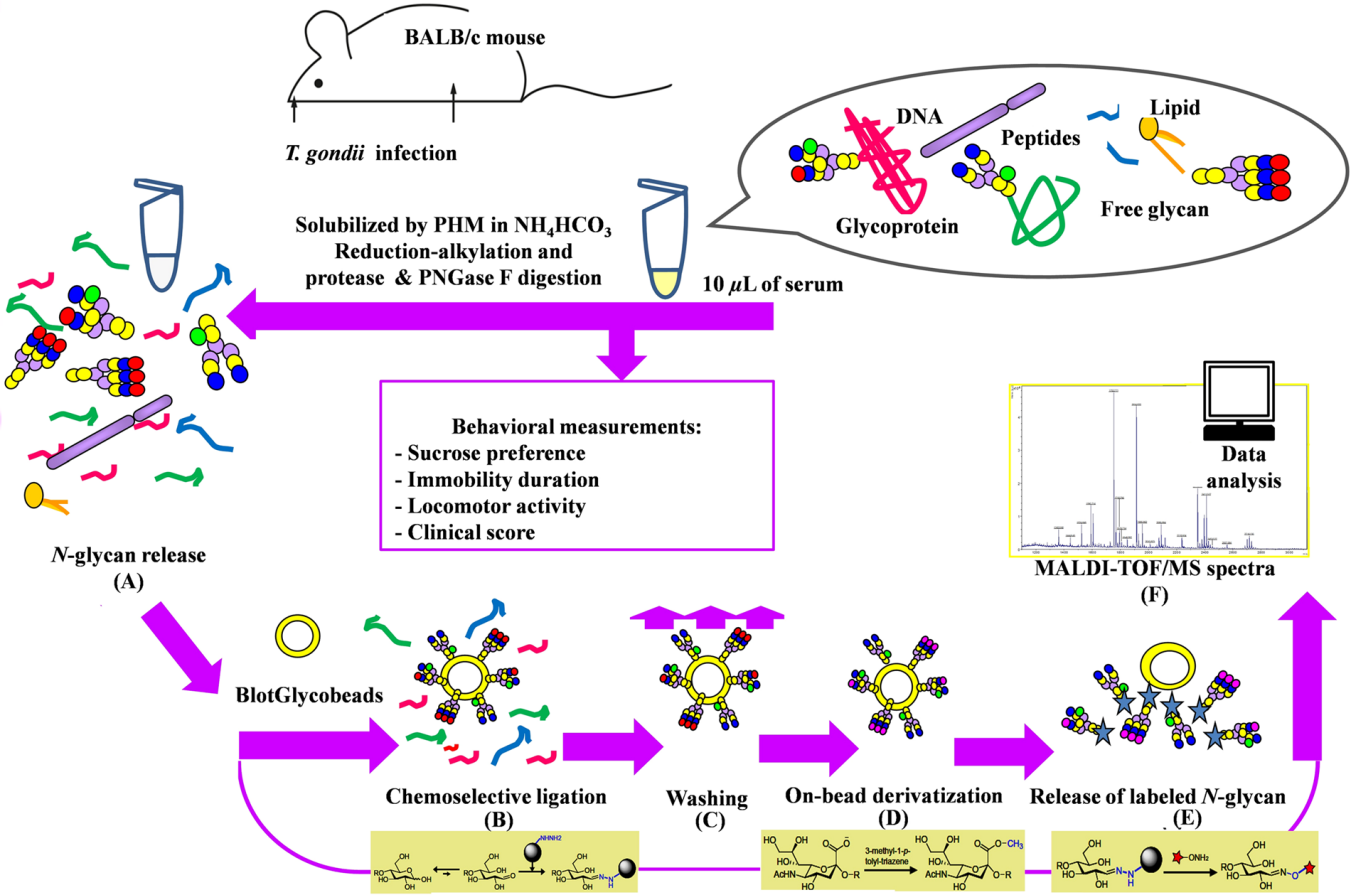
Glycoblotting-based systematic protocol for large-scale glycomics of BALB/c mouse serum samples: **(A)** *N*-glycan release; **(B)** the chemoselective ligation of whole *N*-glycans of serum glycoproteins by “BlotGlyco” beads; **(C)** washing; **(D)** on-bead methylation of sialic acids; **(E)** *trans*-iminization by benzyloxiamine to afford *N*-glycans of the BOA-label; and **(F)** the assessment of total *N*-glycome profiling by MALDI-TOF/MS. The figure had been designed by the authors of this manuscript.

Diagrammatic representation of *N*-glycomics protocol in mouse sera, which is in compliance with the glycoblotting, was shown in Fig. 3. Whole serum *N*-glycans were selectively trapped on glyco-beads to detect *N*-glycan characteristics (steps A~E) using serum glycomics protocols^18,20,24^. The expression of the two principles sialic acid(s) (Neu5Ac-, Neu5Gc-terminal) synthesized by non-mammal cells in glycoproteins might be beneficial for assessing modifications in the regulation of homeostasis, animal behavior, and analysis of *N*-glycomes of BALB/c mice sera. Hence, the listed 18 *N*-glycans structures reported in GlycosuitDB and/or in CFG, were reported^20,24^, the MALDI-TOF/TOF-analysis was not performed.

### Biosynthetic pathway of *N*-glycans during toxoplasmosis

**Figure 4:**
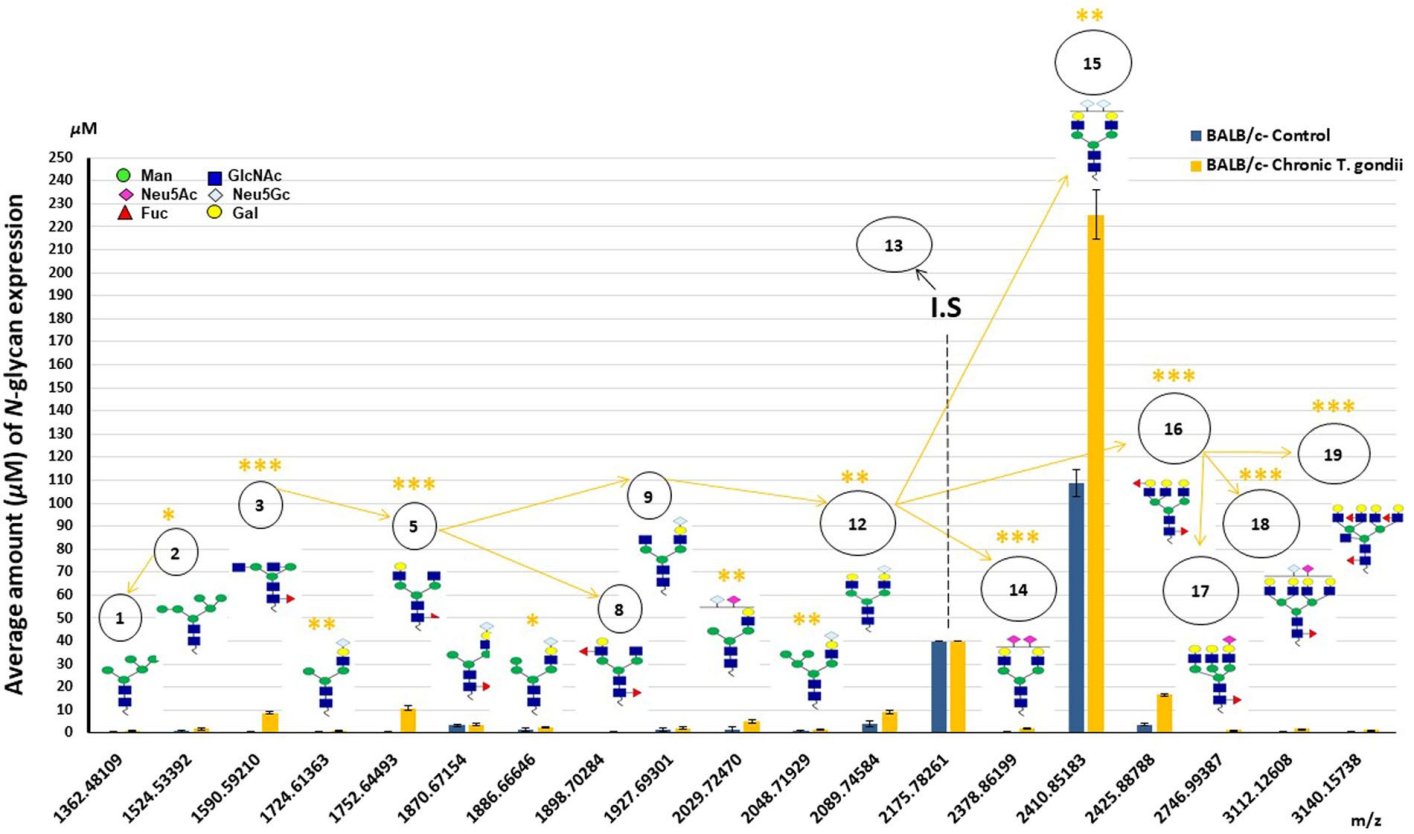
Serum *N*-glycoforms, as provided in experiment-I in the statistical analysis of BALB/c expression concentrations and plausible biosynthetic pathways. Two groups comprised experiment-I: [BALB/c-control, and BALB/c- exposed to chronic *T. gondii* “40 dpi”]. Peak # 13 recognized at *m/z* 2176 indicates internal standard (I.S), [(Hex)2 (HexNAc)2 (Neu5Ac)2 + (Man)3 (GlcNAc)1], and the final concentration was prepared up to 40 *μ*M.

**Figure 5:**
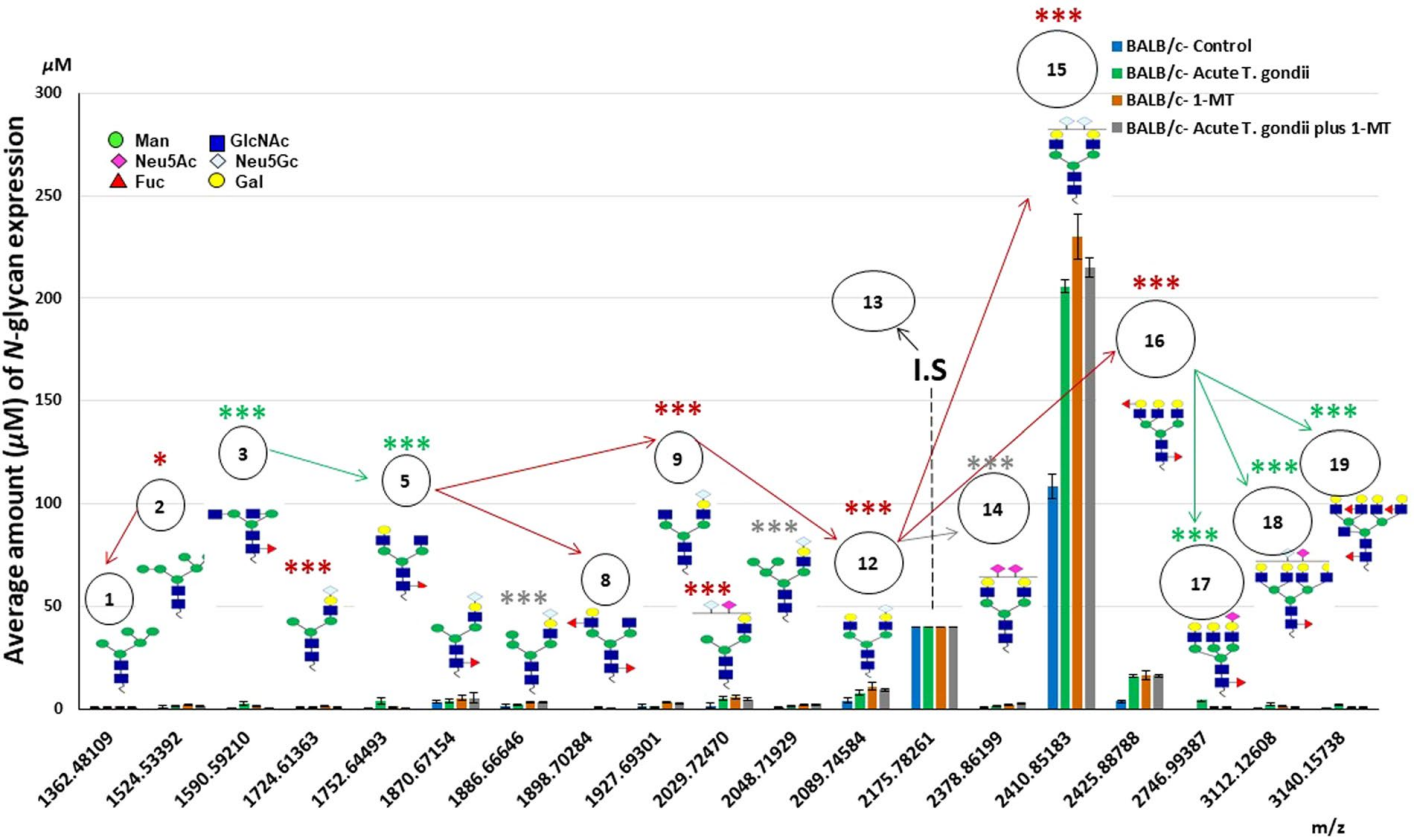
Serum *N*-glycoforms, as provided in experiment-II in the statistical analysis of BALB/c expression concentrations and plausible biosynthetic pathways. Experiment-II [BALB/c-control, BALB/c- exposed to acute *T. gondii* “10 dpi”, and BALB/c- treated with 1-MT after exposure to acute *T. gondii*]. Peak # 13 recognized at *m/z* 2176 indicates internal standard (I.S), [(Hex)2 (HexNAc)2 (Neu5Ac)2 + (Man)3 (GlcNAc)1], and the final concentration was prepared up to 40 *μ*M. 1-MT: 1-methyl tryptophan.

**Figure 6:**
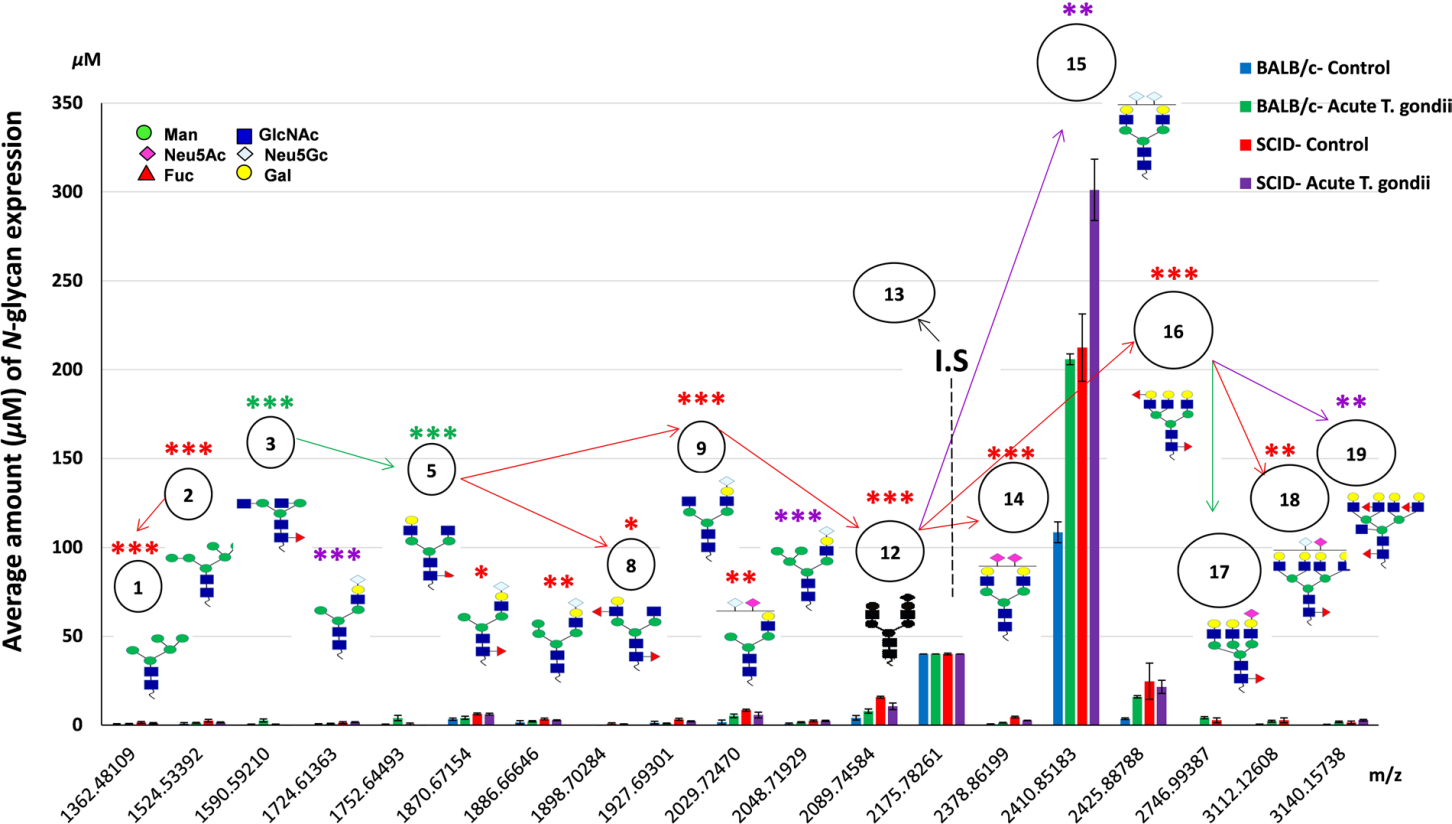
Serum *N*-glycoforms, as provided in experiment-III in the statistical analysis of BALB/c expression concentrations and plausible biosynthetic pathways. Experiment -III [BALB/c-control, BALB/c- exposed to acute *T. gondii* “10 dpi”, SCID-control, and SCID-exposed to acute *T. gondii* “10 dpi”]. Peak # 13 recognized at *m/z* 2176 indicates the internal standard (I.S), [(Hex)2 (HexNAc)2 (Neu5Ac)2 + (Man)3 (GlcNAc)1], and the final concentration was prepared up to 40 *μ*M. SCID, severe combined immunodeficiency.

As anticipated, the more elongated and branched *N*-glycan is the normal pathway of its precursor by the addition of sugar particles through the biosynthetic relevance. For instance, as shown by the arrows in Figs. 4, 5 and 6, in experiment I, II, and III, respectively, Peak # 9 was highly expressed (Tables 1, 2, in experiment I~III). This peak seemed to be the *N*-glycan precursor terminated with Neu5Gc residue. It may be a result of a compensatory mechanism of physiological stress in chronic infection or due to enhanced immunity through anti-depressant drug 1-MT administration in acute infection compared to control. It was considered a counterpart of the *N*-glycan detected in Peak # 12 after adding *Gal*-terminal and followed by Peak # 18 contained sialic acid residue. The latest peak resulted from glycosylation modifications of Peak # 16 by converting tri-into tetra-antennary glycan and exceeding of (1 × Neu5Ac, and supplementary sugar molecules). Overall, it is clearly demonstrated in the mass spectra of animals. However, Peak # 12 was apportioned in all experiments to be counterparts of the Peak # 14, via the inter-conversion between Neu5Gc and Neu5Ac and exceeding of Neu5Ac-residue.

**Table 1:** Estimated compositions of 18 major *N*-glycans of BALB/c mouse serum in experiment-I, II, and III.

Peak #	Composition	Type
**1**^(a)^	(Hex)2 + (Man)3 (GlcNAc)2	High-mannose
**2**^(a)^	(Hex)3 + (Man)3 (GlcNAc)2	High-mannose
**3**^(a)^	(HexNAc)2 (Deoxyhexose)1 + (Man)3 (GlcNAc)2	Hybrid
**4**^(b)^	(Hex)1 (HexNAc)1 (NeuGc)1 + (Man)3 (GlcNAc)2	Hybrid
**5**^(a)^	(Hex)1 (HexNAc)2 (Deoxyhexose)1 + (Man)3 (GlcNAc)2	Complex
**6**^(c)^	(Hex)1 (HexNAc)1 (Deoxyhexose)1 (NeuGc)1 + (Man)3 (GlcNAc)2	Hybrid
**7**^(b)^	(Hex)2 (HexNAc)1 (NeuGc)1 + (Man)3 (GlcNAc)2	Hybrid
**8**^(a)^	(Hex)1 (HexNAc)2 (Deoxyhexose)2 + (Man)3 (GlcNAc)2	Complex
**9**^(a)^	(Hex)1 (HexNAc)2 (NeuGc)1 + (Man)3 (GlcNAc)2	Complex
**10**^(b)^	(Hex)1 (HexNAc)1 (NeuAc)1 (NeuGc)1 + (Man)3 (GlcNAc)2	Hybrid
**11**^(a)^	(Hex)3 (HexNAc)1 (NeuGc)1 + (Man)3 (GlcNAc)2	Hybrid
**12**^(a)^	(Hex)2 (HexNAc)2 (NeuGc)1 + (Man)3 (GlcNAc)	Complex
**13**	(Hex)2 (HexNAc)2 (NeuAc)2 + (Man)3 (GlcNAc)1	I.S
**14**^**(d)**^	(Hex)2 (HexNAc)2 (NeuAc)2 + (Man)3 (GlcNAc)2	Complex
**15**^(a)^	(Hex)2 (HexNAc)2 (NeuGc)2 + (Man)3 (GlcNAc)2	Complex
**16**^(c)^	(Hex)3 (HexNAc)3 (Deoxyhexose)2 + (Man)3 (GlcNAc)2	Complex
**17**^(e)^	(Hex)4 (HexNAc)3 (Deoxyhexose)1 (NeuAc)1 + (Man)3 (GlcNAc)2	Complex
**18**^(c)^	(Hex)5 (HexNAc)4 (Deoxyhexose)1 (NeuAc)1 + (Man)3 (GlcNAc)2	Complex
**19**^(e)^	(Hex)4 (HexNAc)5 (Deoxyhexose)3 + (Man)3 (GlcNAc)2	Complex

^a)^ Glycosuit and/or CFG database, BALB/c mouse glycan structures could be recognized in serum, ^(b)^ “no source”, ^(c)^ not recognized in mice (reported in human), ^(d)^ unidentified in mice (reported in rat), and ^(e)^ “not categorized”. Peak #13 is an internal (quantified) standard spike. GlycoMod (ExPASy proteomics server, Swiss Institute of Bioinformatics), (http://www.expasy.ch/tools/glycomod/), has been made to detect the compositional annotation and estimated structures. However, the non-reported CFG database structures were detected through (http://www.functionalglycomics.org). Two high-mannose, six hybrids, and ten complex *N*-glycans were identified in mice serum. HexNAc, *N*-acetylhexosamine (GlcNAc, *N*-acetylglucoseamine [blue square] or GalNAc, *N*-acetylgalactoseamine [yellow square] depends on the description); Deoxyhexose, fucose [red triangle]; Hex, hexose (Mannose [green circle], and galactose [yellow circle] depend on the description); Neu5Ac, 5-*N*-acetylneuraminic acid [purple diamond]; and Neu5Gc, 5-*N*-glycolylneuraminic acid [white diamond].I.S, internal standard.

**Table 2:** The significance levels (mean ± SD) of 18 major *N*-glycans in mice serum in experiment-I, II, and III.

Treatment No.	1	2	3	4	5	6	7	1,2	1,3,4,5	1,3,6,7
Peak #	BALB/c –Control (*μ*M ± SD)	BALB/c – *T. gondii* (40 dpi) (*μ*M ± SD)	BALB/c – *T. gondii* (10 dpi) (*μ*M ± SD)	BALB/c – 1-MT (*μ*M ± SD)	BALB/c – *T. gondii* (10 dpi) plus 1-MT (*μ*M ± SD)	SCID-Control (*μ*M ± SD)	SCID-*T. gondii* (10 dpi) (*μ*M ± SD)	Exp. I *P*-value	Exp. II *P*-value	Exp. III *P*-value
**1**^(a)^	0.52 ± 0.3	**1.00** ± **0.34**	0.83 ± 0.13	**1.06** ± **0.2**	0.72 ± 0.11	**1.51** ± 0.5***	1.07 ± 0.36	**0.058**	**0.137**	**0.000**
**2**^(a)^	0.87 ± 0.54	**1.84** ± 0.48*	1.29 ± 0.24	**2.01** ± 0.31*	1.41 ± 0.09	**2.48** ± 0.76***	1.5 ± 0.24	**0.025**	**0.023**	**0.000**
**3**^(a)^	0.38 ± 0.25	**8.88** ± 0.63***	**2.60** ± 0.85***	1.39 ± 0.26	0.38 ± 0.07	0.45 ± 0.15	………	**0.000**	**0.000**	**0.000**
**4**^(b)^	0.48 ± 0.3	**0.96** ± 0.10*	0.92 ± 0.16	**1.2** ± 0.15***	1.00 ± 0.03	1.41 ± 0.50	**1.63** ± 0.22***	**0.012**	**0.000**	**0.001**
**5**^(a)^	0.58 ± 0.06	**10.89** ± 0.91***	**3.97** ± 1.47***	1.1 ± 0.21	0.41 ± 0.08	0.69 ± 0.50	………	**0.000**	**0.000**	**0.000**
**6**^(c)^	3.36 ± 0.6	**3.67** ± **0.39**	4.08 ± 0.92	5.30 ± 1.32	**5.33** ± **2.3**	**6.36** ± 0.50*	6.15 ± 0.47	**0.737**	**0.109**	**0.050**
**7**^(b)^	1.51 ± 0.92	**2.53** ± 0.2*	2.09 ± 0.36	3.22 ± 0.4	**3.25** ± 0.16***	**3.43** ± 0.50**	2.74 ± 0.21	**0.046**	**0.000**	**0.004**
**8**^(a)^	………	**0.67** ± **0.02**	………	**0.7** ± **0.11**	………	**0.86** ± 0.50*	0.66 ± 0.03			**0.028**
**9**^(a)^	1.37 ± 0.78	**2.22** ± 0.35**	1.02 ± 0.19	**3.36** ± 0.4***	2.41 ± 0.23	**3.25** ± 0.50***	2.14 ± 0.26	**0.005**	**0.000**	**0.000**
**10**^(b)^	1.62 ± 1.29	**5.26** ± 0.78**	5.2 ± 1.05	**5.56** ± 1.01***	4.75 ± 0.51	**8.42** ± 0.50**	5.65 ± 1.56	**0.002**	**0.001**	**0.002**
**11**^(a)^	0.73 ± 0.46	**1.71** ± 0.22**	1.61 ± 0.33	2.08 ± 0.29	**2.28** ± 0.24***	2.4 ± 0.50	**2.41** ± 0.35***	**0.004**	**0.000**	**0.001**
**12**^(a)^	4.12 ± 1.29	**9.15** ± 0.84**	7.87 ± 1.23	**10.82** ± 1.81***	9.35 ± 0.59	**15.71** ± 0.50***	10.6 ± 1.82	**0.004**	**0.000**	**0.000**
**14**^**(d)**^	0.7 ± 0.058	**2.00** ± 0.21***	1.31 ± 0.17	2.06 ± 0.21	**2.85** ± 0.21***	**4.51** ± 0.50***	2.61 ± 0.1	**0.000**	**0.000**	**0.000**
**15**^(a)^	108.5 ± 5.89	**225.3** ± 10.68*	205.84 ± 3.00	**229.87** ± 11.00***	215.31 ± 4.71	212.44 ± 18.92	**301.18** ± 17.3**	**0.014**	**0.001**	**0.002**
**16**^(c)^	3.6 ± 0.47	**16.6** ± 0.6***	15.91 ± 0.66	**16.47** ± 2.28***	16.11 ± 0.82	**24.63** ± 10.21***	21.51 ± 3.71	**0.000**	**0.000**	**0.001**
**17**^(e)^	………	**1.3** ± **0.07**	**4.21** ± 0.61***	1.06 ± 0.18	………	2.69 ± 1.41	………		**0.000**	**0.077**
**18**^(c)^	0.43 ± 0.11	**1.75** ± 0.18***	**2.19** ± 0.5***	1.12 ± 0.25	1.07 ± 0.21	**2.69** ± 1.41**	………	**0.000**	**0.000**	**0.009**
**19**^(e)^	0.31 ± 0.18	**1.27** ± 0.15***	**1.84** ± 0.38***	0.97 ± 0.18	0.66 ± 0.13	1.55 ± 0.75	**2.76** ± 0.52**	**0.000**	**0.000**	**0.005**

Demonstrating the importance of *N*-glycan, which appears in the *t*-student test, for the largest expression of the serum N-glycoforms in all mice groups, by statistical analytics. Experiment-I [BALB/c-control, and BALB/c- exposed to chronic *T. gondii*], experiment-II [BALB/c-control, BALB/c- exposed to acute *T. gondii*, 1-MT, and BALB/c- treated with 1-MT after exposure to acute *T. gondii*], experiment-III [BALB/c-control, BALB/c- exposed to acute *T. gondii*, SCID-control, and SCID-exposed to acute *T. gondii*]. (“–”) no detection has been made. The meaning level of the asterisk “***”, *P* ≤  0.001, the asterisk “**”, *P* ≤  0.01 and the asterisk “*”, *P* ≤  0.05. 1-MT: 1-methyl tryptophan; Exp.: experiment, SCID: severe combined immune deficiency, and SD: standard deviation.

Therefore, our study was designed to examine serum *N*-glycan profiles in three experiments as following:

#### Control vs chronic infection of *T. gondii*

Sixteen and eighteen key serum *N*-glycans in control-and infected-BALB/c mice (40 dpi), respectively were assayed via MALDI-TOF/MS (see Fig. S1 in Supplementary Information). Separated MS spectra showed *N*-glycan in control as well as *T. gondii* chronically infected mice (see Figs. S4, S5 in Supplementary Information). 13 peaks (72.2%) were observed in GlycosuitDB and the remaining peaks were detectable in CFG database. Almost all the detected *N*-glycan structures were originated in *Mus Musculus* sera, particularly mouse serum. All the mass spectra of different treatments were highly reproducible with sensible oscillations in their expression. It is worthy to mention that anonymous mass expressions/peaks with significant intensities might exist either because the trapped free-reducing oligosaccharides through the glycoblotting or did not result in reproducible peaks for the quantitative analysis of almost study experiments. Accordingly, unidentified peaks were not considered valid for the statistical analysis. Therefore, the metabolic pathways of these peaks and their significances were not addressed. Sugar structural contents and masses of serum *N*-glycans in mouse sera glycoproteins (Table 1) were represented by *N*-glycan amount (*µ*M ± SD) and their significance levels were calculated (see Table 2). Moreover, detected ions of observed *m/z* values; generated after MALDI-TOF/MS analysis; exceeded comparatively than the ExPasy *m/z* values. The reason is as follows: i) *trans*-amination reaction through BOA-labeled *N*-glycans (105 *m/z*), ii) generation of Na-adducting (23 *m/z*) in laser release through the shooting process to accomplish the mass spectral-analysis and, iii) sialic acid esterification for each methylation grouping (14 *m/z*), (see Table S1 in Supplementary Information). The ions peaks of serum *N*-glycome expressions of mice were confirmed to be ≥80%. The alterations of the expression levels of *N*-glycan profiles together with their plausible biosynthetic pathway mentioned as micromolar (i.e., 100 *μ*g of glycoproteins) whether in experimental-or in control mice (see Fig. 4). Herein, mice’s *N*-glycans types divided into 2 high-mannose (13, 11%), 6 hybrid (38, 33%), and 8, 10 complex types (50, 56%), in control and in chronic-*T. gondii* infection respectively, shown in Table 3. Moreover, glycotyping-analysis of selected *N*-glycans high-mannose, core-fucosylated, bisecting, and (tri/tetra)-antennary *N*-glycans expressions confirmed in the mass spectra expression/peaks of experimental mice sera. Also, this kind of analysis revealed a novelty of the *N*-glycan pathway in those experimental mice (Table 4). The sialylated glycomic results in Peak # 14 at *m/z* 2378 (2 × Neu5Ac) showed a robust negative correlation with the sucrose preference ratio (−0.558**) and a weak positive correlation with the walking abilities (0.484*) of infected mice (40 dpi). However, Peak # 15 at *m/z* 2410 (2 × Neu5Gc) manifest opposite correlation coefficients to the sucrose preference ratio and the walking abilities (0.303* and −0.703***, respectively), (shown in Table 5A). Correlation analysis for the rest of peaks with the behavioral data was also calculated (see Table S5 in Supplementary Information).

**Table 3:** Ratio of *N*-glycan of BALB/c mouse serum in experiments I, II and III.

*N*-glycan type %	BALB/c-CNT	BALB/c *T. gondii* (40 dpi)	BALB/c *T. gondii* (10 dpi)	BALB/c 1-MT	1-MT + BALB/c *T. gondii* (10 dpi)	SCID-CNT	SCID *T. gondii* (10 dpi)
**High-man%**	13%	11%	12%	11%	13%	11%	14%
**Hybrid%**	38%	33%	35%	33%	38%	33%	36%
**Complex%**	50%	56%	53%	56%	50%	56%	50%

Experiment-I [BALB/c-control, and BALB/c- exposed to chronic *T. gondii*], experiment-II [BALB/c-control, BALB/c- exposed to acute *T. gondii*, 1-MT, and BALB/c- treated with 1-MT after exposure to acute *T. gondii*], experiment-III [BALB/c-control, BALB/c- exposed to acute *T. gondii*, SCID-control, and SCID-exposed to acute *T. gondii*]. 1-MT: 1-methyl tryptophan; CNT: control; SCID: severe combined immune deficiency, and SD: standard deviation.

**Table 4:** Glycotyping analysis (mean ± SD) of high-mannose, mono-fucosylated, and mono-, di-, tri-,tetra-sialylated, bisecting and bi-,tri-,tetra-antennary *N*-glycan% of BALB/c mouse serum in experiment I, II and III.

Glycotyping	BALB/c-CNT	*T. gondii* (40 dpi)	*T. gondii* (10 dpi)	1-MT	1-MT + *T. gondii* (10 dpi)	SCID-CNT	SCID *T. gondii* (10 dpi)
**High-Man**	1.39 ± 0.03	2.84 ± 0.82	2.12 ± 0.36	3.07 ± 0.51	2.13 ± 0.20	3.99 ± 0.54	2.57 ± 0.59
**Mono-Fuc**	4.64 ± 2.40	26.48 ± 1.38	17.05 ± 3.59	9.97 ± 1.66	8.19 ± 2.18	12.88 ± 2.46	6.15 ± 2.91
**Di-Fuc**	2.7 ± 1.84	17.27 ± 0.59	6.63 ± 1.31	2.19 ± 0.02	0.57 ± 0.003	20.57 ± 5.13	22.17 ± 5.73
**Tri-Fuc**	0.23 ± 0.21	1.27 ± 0.15	1.84 ± 0.38	0.97 ± 0.18	0.66 ± 0.13	1.55 ± 0.26	2.76 ± 0.52
**Sial1**	11.52 ± 2.5	23.28 ± 1.69	23.99 ± 3.33	28.15 ± 4.30	25.86 ± 1.53	2.93 ± 0.31	25.67 ± 3.75
**Sial2**	83.27 ± 5.7	232.56 ± 0.77	211.83 ± 4.82	237.49 ± 1.20	221.77 ± 3.05	1.86 ± 0.35	309.44 ± 1.50
**Bisecting**	0.23 ± 0.005	1.27 ± 0.15	1.84 ± 0.38	0.97 ± 0.09	0.66 ± 0.13	1.55 ± 0.26	2.76 ± 0.52
**Bi-anten**	88.51 ± 5.98	259.11 ± 10.15	222.1 ± 0.009	249.3 ± 3.77	229.98 ± 0.90	243.16 ± 23.49	317.18 ± 1.72
**Tri-anten**	2.69 ± 0.3	17.89 ± 0.63	13.75 ± 0.86	14.23 ± 0.43	10.83 ± 0.33	36.51 ± 4.31	21.51 ± 1.86
**Tetra-anten**	0.55 ± 0.009	3.02 ± 0.23	4.03 ± 2.43	2.09 ± 0.002	1.73 ± 0.009	6.70 ± 1.17	2.76 ± 1.96

Experiment-I [BALB/c-control, and BALB/c- exposed to chronic *T. gondii*], experiment-II [BALB/c-control, BALB/c- exposed to acute *T. gondii*, 1-MT, and BALB/c- treated with 1-MT after exposure to acute *T. gondii*], experiment-III [BALB/c-control, BALB/c- exposed to acute *T. gondii*, SCID-control, and SCID-exposed to acute *T. gondii*]. 1-MT: 1-methyl tryptophan; CNT: control; SCID: severe combined immune deficiency, and SD: standard deviation.

**Table 5:** Pearson’s correlation coefficients for % of sucrose preference, immobility duration, line crossing, and the levels of serum *N*-glycans (Peak# s 14 and 15) in *T. gondii* infected mice.

*N*-glycans Peak #	Sucrose preference (r)	Immobility duration (r)	Line crossing (r)	Clinical score (r)
**A**				
14	−0.558**	0.176	0.484*	
15	0.303	−0.896***	−0.703***	
**B**				
14	0.696**	0.845***	0.497*	0.159
15	−0.389*	0.096	0.032	0.920***
**C**				
14	0.766***	−0.580**	−0.141	−0.168
15	−0.210	0.026	−0.163	0.439*
**D**				
14	0.993***	0.895**	0.961***	−0.867***
15	−0.968***	−0.673**	−0.797**	0.988***

Each value represent Pearson’s correlation coefficients *** [r] = 0.7, strong correlation, ** [r] = 0.5–0.7, moderately to strong correlation, and * [r] = 0.3–0.5, weak to moderate correlation. BALB/c- exposed to chronic *T. gondii*], (A); BALB/c- exposed to acute *T. gondii*, (B); BALB/c- treated with 1-methyl tryptophan (1-MT) after exposure to acute *T. gondii*], (C); and severe combined immune deficiency (SCID)-exposed to acute *T. gondii*], (D). SD: standard deviation.

#### Control vs acute infection of *T. gondii*, 1-MT alone, then acute infection/1-MT

Likewise, 16, 17, 18 and 16 key serum *N*-glycans in (control, infected BALB/c mice (10 dpi), administrated with 1-MT and the infected BALB/c mice group (10 dpi) which treated with 1-MT, respectively) were detected (see Fig. S2 in Supplementary Information). Other separated MS spectra of acute *T. gondi* in mice are represented (see Fig. S6 in Supplementary Information). Moreover, MS spectra of 1-MT therapy and infected BALB/c after treatment 1-MT are represented (see Figs. S7, S8 in Supplementary Information). Also, in experiment-I, the molecular masses and the sugar compositions are shown in (Table 1) are represented by *N*-glycan amount and their significance level (Table 2). However, the alteration of expressed *N*-glycans and their plausible biosynthetic pathway in mouse serum were illustrated (see Fig. 5). The serum *N*-glycomic types divided into 2 high-mannose (13, 12, 11 and 13%), 6 hybrid (38, 35, 33 and 38%), and 8, 9, 10 and 8 complex types (50, 53, 56 and 50%), in control, acute infection, 1-MT and acute infection treated with 1-MT, respectively, shown in Table 3. The glycotyping-analysis revealed a specialty of the biosynthetic pathway of *N*-glycan in experimental mice (Table 4). Interestingly as shown in Table 5B, a positive correlation was manifested with sucrose preference ratio (0.696**), immobility duration (0.845***), and walking abilities (0.497*) of infected mice (10 dpi) at Peak # 14. However, Peak # 15 had a weak negative correlation to the sucrose preference (−0.389*) and a strong positive correlation to the clinical score (0.920***). After 1-MT therapy (Table 5C), Peak # 14 was shifted to a moderate negative correlation of the immobility duration (−0.580**) but Peak # 15 seemed to be of the same correlation coefficients of infected mice (10 dpi). Other correlation analysis has been detected for the rest of the peaks (see Table S3 and S4 in Supplementary Information).

#### Control vs acute infection of *T. gondii*, SCID alone, then acute infection/SCID

Similarity, 16, 18, 18 and 14 key serum *N*-glycans in control, infected BALB/c mice (10 dpi), SCID and infected SCID mice (10 dpi) were observed (see Fig. S3 in Supplementary Information). Other separated MS spectra of SCID mice and infected SCID (10 dpi) are represented (see Figs. S9, S10 in Supplementary Information). Also, in experiment-I,-II, the molecular masses, as well as the sugar compositions of mouse serum glycoproteins (Table 1), are represented by *N*-glycan amount and their significance expression levels (in Table 2). While the alteration of the *N*-glycans expressions and their plausible biosynthetic pathway in mouse serum represented in Fig. 6. The serum *N*-glycomic types divided into 2 high-mannose (11, 12, 11 and 11%), 6, 6, 6, 5 hybrid (33, 35, 33 and 43%), and 8, 9, 10 and 7 complex types (33, 35, 43 and 43%), in control, BALB/c -acute infection, as well as healthy and infected SCID mice, shown in Table 3. The glycotyping-analysis showed a specialty of the biosynthetic pathway of the *N*-glycome profiles in experimental mice (Table 4). Surprisingly, positive correlation showed up with the sucrose preference ratio (0.993***), immobility duration (0.895**) and walking abilities (0.961***) but a strong negative correlation to the clinical score (−0.867***) of infected SCID mice (10 dpi) at peak 14. However, Peak # 15 showed opposite correlation coefficients (shown in Table 5D). The other correlated peaks have been detected (see Table S5 in Supplementary Information).

Our result for three experiments would help to create novel biomarkers of depression, represented in Peak # 14 and Peak # 15 (Table 6).

**Table 6:** Specific peaks (*μ*M ± SD) of serum *N*-glycan as biomarkers of depressive-like behaviors in mice.

	Peak #	*m/z*	ExPasy (MW)	Composition	Type	Source	Group.1	Group.2	Group.3	Group.4
Experiment-I	**14**	2378.86	2222.78	(Hex)2 (HexNAc)2 (NeuAc)2 + (Man)3 (GlcNAc)2	Complex	UniCarbKB	0.7 ± 0.06	**2.00** ± 0.2***		
Experiment-II							0.7 ± 0.06	1.31 ± 0.2	2.1 ± 0.21	**2.8** ± 0.21***
Experiment-III							0.7 ± 0.06	1.31 ± 0.2	**4.51** ± 0.5***	2.61 ± 0.1
Experiment-I	**15**	2410.85	2254.77	(Hex)2 (HexNAc)2 (NeuGc)2 + (Man)3 (GlcNAc)2	Complex	UniCarbKB	108.5 ± 5.9	**225.3** ± 10.7*		
Experiment-II							108.5 ± 5.9	205.84 ± 3.0	**229.9** ± 11.0***	215.31 ± 4.7
Experiment-III							108.5 ± 5.9	205.9 ± 3.0	212.4 ± 19	**301.2** ± 17.3***

Based on the protein concentration in the BALB/c experiment-II and the glycosuit dataset, 100 *μ*g proteins for *N*-glycans and 10 *μ*L of serum glycoproteins have been used. Peak #s **14**, **15** can be distinguished among the three experiments. Experiment-I [group.1, BALB/c-control, and group.2, BALB/c- exposed to chronic *T. gondii*], experiment-II [group.1, BALB/c-control, group.2, BALB/c- exposed to acute *T*. gondii, group.3, 1-methyl tryptophan (1-MT), and group.4, BALB/c- handled with 1-MT 1-MT after exposure to acute *T. gondii*], and experiment-III [group.1, BALB/c-control, group.2, BALB/c- exposed to acute *T. gondii*, group.3, severe combined immunodeficiency (SCID)-control, and group.4, SCID-exposed to acute *T. gondii*]. GlycoMod (ExPASy proteomics server, Swiss Institute of Bioinformatics), (http://www.expasy.ch/tools/glycomod/), has been made to detect the compositional annotation and estimated structures. Glycan cartoon was assembled through the typical consortium for functional nomenclature, i.e., GlcNAc, *N*-acetylglucoseamine [blue square]; Hex, hexose (Mannose [green circle], galactose [yellow circle]; and Neu5Ac, 5-*N*-acetylneuraminic acid [purple diamond]. The asterisk “***” showed a significance level and *P* ≤  0.001, “*”, *P* ≤ 0.05). SD: standard deviation.

## Discussion

Depression is a multi-factorial illness. Here, we showed that depression-related behaviors are associated with enhancement of immune response during *T. gondii* infection. *T. gondii* can cause nervous disorders results from the influence of host behavior. Under our setting in experiment-I, mice chronically infected with *T. gondii* exhibited neither anhedonic-nor despair-like behaviors (Figs. 1A, 2A). In experiment II; 1-MT reduced the sickness, anhedonic and despair-like behaviors at 10 dpi with *T. gondii* (Figs. 1B, 2B). Recently, we showed that 1-_DL_-MT exhibited anti-toxoplasma activity *in vivo* and *in vitro*^1^. Therefore, 1-MT treatment successively improved clinical score (Fig. 2D), increased sucrose preference and reduced immobility duration in FST (Fig. 1B) along with improved locomotion (Fig. 2B) comparable to control.

### Glycan and depression in infected immunocompetent mice of toxoplasmosis (10-, and 40 dpi)

We aimed to investigate whether the *N*-glycans expressions were altered in chronic/acute *T. gondii* infection (experiment-I/II). The estimated composition of *N*-glycans shown in Table 1 and the significance levels showed in Table 2. MS spectra in experiment I, II were represented in Figs. S1, S2 in Supplementary Information, respectively. MALDI-TOF/MS *N*-glycomics result of mice sera was significantly altered in acute and in chronic *T. gondii* infections compared to control mice. The reason might be related to the underlying mechanism of immunostimulation with the family dynamics, and the post-transitional glycosylation of glycoproteins to modulate their biological functions^30^. Herein, our finding indicated that the expression levels of *N*-glycomics have distinctive *sial*-terminal(s) either Neu5Ac or Neu5Gc in mice sera. It was common to notice that the sialylated *N*-glycans were altered in *T. gondii* infection and represented by peaks. Thusly, it was not a surprise that terminated-*sial*-terminal(s) *N*-glycans was found in mice, supporting the fact that this animal naturally produces both sialic acids (Fig. S1, S2 in Supplementary Information, in experiment I, II, respectively). It depends upon the activity of the gene(s) encoded the CMP-Neu5Ac Hydroxylase enzyme, which converts Neu5Ac into Neu5Gc^31^. However, the most interesting finding is consigning glycans to simply differentiate the normal, infected with chronic/acute *T. gondii* infection, and then treated mouse with antidepressant medication. Neu5Ac regulates the biological functions of outermost anion layer of cellular surfaces in human and/or animal; For instance, cell-contact-mediated repulsion, physiological condition of kidneys, maternal immune responses, anti-aging for the long life, cognitive behaviors, and attending as an inhibiting factor of bacteria, viruses, and toxins^32^. Consequently, the findings revealed that the biological significance of Neu5Ac in modulating the action of the central nervous system such as the consciousness, cognitive behaviors, reflexes, and efficiency of the immunity.

### Impact of 1-MT therapy of toxoplasmosis on glycomics

The *N*-glycan profiles bearing with Neu5Ac-terminal(s) clearly represented higher expression in mice group infected with chronic-, acute-*T. gondii* infection or those treated with an antidepressant drug, (see Tables 1, 2) and (Fig. S1 “gr.2”, Fig. S2 “gr.4”, in Supplementary Information, of experiment I, II, respectively) than in control group. The Neu5Ac-terminal showed a shifting of animals to the normal behavioral displays against parasitic infection (Fig. 1A, Fig. 1B in experiment I, II, respectively). The *N*-glycan bearing with Neu5Ac, was clearly expressed in the mice group infected with chronic, acute *T. gondii* (experiment I, II) compared to control. Moreover, the Neu5Ac-terminated *N*-glycan was started in Peak # 10, which referred to a basic alarm of normal immune response. We noticed that Peak #s 14, 17, 18 at *m/z* 2378 (2 × Neu5Ac), 2746 (1 × Neu5Ac), and 3112 (1 × Neu5Ac) terminals, respectively, were designated to give typical model of the homeostasis normality. Particularly, Peak # 14 that was of high *N*-glycans expression in the mice treated with 1-MT after the acute *T. gondii* infection, due to the effect of such immune-stimulant drug on the animal immune balance or its anti-toxoplasma activity^6^. The peaks terminated by Neu5Ac were expressed to define abundantly the capability of the mouse’s immune system to regulate the homeostasis. Therefore, sialylated *N*-glycan bearing those Neu5Ac-terminal(s) was abundantly expressed and can differentiate between the infected (whether 10 dpi or 40 dpi), and the non-infected mice of toxoplasmosis. However, Neu5Gc has enzymatically resulted in the addition of a hydroxyl group by Neu5Ac. It might has an epigenetical role for accommodating animals to all the dynamic changes of acquired infections to provoke the immune response -typically- found as an endogenous form of sialic acid^33^. The Neu5Gc-terminated *N*-glycans were detected among mice groups, especially Peak #s 4~12 (Tables 1, 2 and Figs. 4, 5, for experiments I, II, respectively). We believed that the abundant usage of Neu5Gc-terminated *N*-glycans, (Peak # 15 at *m/z* 2410), in common with the putative baseline of the standard mechanism of immune defense (Neu5Ac-terminal, Peak # 10) were probably found in MS spectra to manage, as possible, the *T. gondii* infection in mice. This mechanistic pathway may be displayed again after exposing mice to 1-MT therapy to modulate the acute infection with *T. gondii*. Taking altogether, it indicates the biological role of Neu5Gc signals of immune resistance to acclimate toxoplasmosis. It was probably related to the anhedonic- besides despair-like behavioral condition, and consequently mouse reduced significantly the appetite in addition to the responsiveness/awareness (*P*<0.001 each, Fig. 1B in experiment II). However, the substantiation of peaks detected afterwards proposes the wide-ranging success of homeostatic balance/regulation to modulate the immune system and the role of epigenomic plasticity. This might link to the immune responsiveness, along with short vs long-freezing/immobility time “*P*<0.001 each”. It resulted in short vs long-interval depressive-like behavior (Fig. 1A,B in experiment I vs II). It revealed that sickness and depression-related behaviors (anhedonia and immobility in FST) were not different in normality and/or the chronic infection (40 dpi) of BALB/c mice. The genetic resistance might be the reason why BALB/c mice strain adapted *T. gondii* infection; consequently understanding its ability to develop a chronic infection^14^. However, these behaviors were evident in BALB/c mice during acute infection (10 dpi). The statistical analysis (see Table 5C) revealed that the expression level of Peak # 14 at *m/z* 2378, after 1-MT therapy of *T. gondii* (10 dpi), had a moderate negative correlation with the immobility duration (−0.580**). It means that 1-MT improved the fitness of infected mice. Therefore, we need to realize the importance of sialylated *N*-glycans. Particularly, the IgG antibody is considered the main IgGs in serum *N*-glycan of the human/animal. Moreover, IgG has been used in pharmaceutical industries for its specific structural stability of *N*-glycan IgG, along with multi-functional properties like the core-fucosylation, galactosylation, and sialylation^34^. It was reported that the addition of galactose, as well as moieties of sialic acid(s) to IgG *N*-glycans, induces the anti-inflammatory process^35^. Moreover, IgG in cow colostrum was reported to be more abundantly contained Neu5Ac and Neu5Gc than in normal milk^20^. The same authors revealed that it commonly related to the maternal immunity relationship. Thus, we could understand that the glycoproteins/IgGs sialylation was abundantly noticed in order to standardize immune cell functions in different pathological conditions in animal/human sera. This sialylation process was completed through connections with the receptors of Fcγ/FcRn as well as multiple pattern recognition receptors (PRRs) which include sialic acid-binding IgG-receptors (Siglec) on dendritic cells^36^. The epigenetic impact on DN- phenotype and the genetic mutation (epigenetic marks) may be the reason for this adaptation phenomenon; perhaps during such miRNA mutation which interacted with the biosynthetic pathway of glycomics or due to the heritability of glycome composition^37^. The same author reported that this adaptation might resist the influences of any anti-depressant drugs such as tumor necrosis factor and/or amyloid precursor protein that reported in the hippocampus. It might relate to encoding glycans in fibroblast cells after *T. gondii* infection^19^. Therefore, *T. gondii*-induced anhedonia was associated with reduced gene expression for cellular multiplications. Importantly, the transcriptome-analysis of the BALB/c mice’s brain showed that toxoplasma parasite load was correlated with the expressions of genes inducing neurological responses, but not with those involved in the immune functions^38^.

### Glycan and depression in infected immunodeficient mice of toxoplasmosis (10 dpi)

We focused on serum glycan profile during lowered immunity in mice infected with *T. gondii* (experiment-III). Therefore, we decided to understand the sialylation profile of serum *N*-glycans peaks/expressions of SCID mice. SCID mice are genetically related to immunodeficiency syndrome^39^. It may give a clear explanation for the Neu5Ac/Neu5Gc ratio in SCID mice (10 dpi) compared to those obtained from control BALB/c mice. The estimated compositions of *N*-glycan structures of SCID mice, healthy or infected with *T. gondii*, fit those of control BALB/c mice (Table 1). As a result, Fig. S3 in Supplementary Information represented the great alteration of MS spectra of *N*-glycome results of infected SCID with *T. gondii* (10 dpi) in comparison with those healthy-SCID, healthy-BALB/c and BALB/c mice (10 dpi), and under activation of the immune system mice. Moreover, sialylated *N*-glycans (Neu5Ac-, Neu5Gc-terminal) were significantly higher expressed in SCID (see Fig. 6) but getting poorer behavioral reflexes (see Fig. 1C) due to their genetic mutation compared to the BALB/c controls. The behavioral observations of SCID were physically appeared the same as the BALB/c controls (see Figs. 1C, 2C). However, sickness but not depression- related behaviors (anhedonia and immobility in FST) were evident in infected SCID (10 dpi). These SCID mice clinically died after day-10 as expressed in dramatically elevated clinical scoring (see Fig. 2E). It was reported that depressed-patients had fewer natural killer cells as well as T- lymphocytes than in controls^40^. The same group reported that the gene mutation encoding the common gamma chain (Y_c_) was the main cause of sickness signs and fatal toxoplasmic encephalitis in SCID mice. By which, Y_c_ is a protein supported the y the interleukins receptors (such as IL−2,−4,−7,−9,−15 and −21) in the mutation process. It was given by the fact that these interleukins and their receptors may be included in T-and B-cells functions of SCID mice. Therefore, after *T. gondii* post-infection (10 dpi) period, we can understand that SCID mice’s T-cells become anergic and improper B-lymphocyte activation which means that they no longer respond to infection^37^, thus getting adapted. So, these SCID mice may be able to host their immune system but one that may not be functioning properly^41^. The same group revealed that B-cells and T-cells of adaptive immune system in SCID mice were consequently damaged because of the genetic mutation. By which, SCID-mice’s immune system may not activate some components of the complement system and therefore the mice cannot apparently fight *T. gondii* infection. The result clearly indicated that the Peak # 15 at *m/z* 2410 may be a basic line of immune defense in SCID (10 dpi) and had an obvious direct correlation with the clinical score/sickness (0.988***, Table 5D). Equally, the proof of no peaks detected afterword (Fig. 6), may suggest the total failure of homeostatic immune balance. This may result in fatal toxoplasmic encephalitis^9^. However, the significant difference in serum *N*-glycans expressions in mutant SCID mice compared to BALB/c (10 dpi) might be developed during the genetic evolution of SICD to resist any infection *in vivo*^33^. Our findings suggested that SCID mouse was an ideal model to investigate serum glycans profile in immunosuppression and/or *T. gondii* infection.

### Glycotyping analysis

Statistical analyses revealed that the normal pathway of *N*-glycan types initiated by mannose-terminals up to *sial*-terminal(s). For instance, the high-mannose type showed in Peak #s 1, 2 at *m/z* 1362, 1524, respectively was clearly expressed (Tables 1, 2; *P*<0.05, *P*<0.001, in experiment I, II, respectively) in mice infected with chronic/acute *T. gondii* than the expression in controls. It indicated the downregulation of *exo*-mannosidase enzyme which has an important role for the trimming process of the multi-mannose residue(s) of high-mannose *N*-glycan type; an essential simple substrate for the coming synthetic processes of the mature/complex *N*-glycan type. Thusly, complex glycan like (tetra-antennary, Peak # 18) was expressed significantly in chronic/acute *T. gondii* than control. Glycotyping-analysis (see Table 4 in experiment I, II, and III) of the selected *N*-glycans indicated that “matured glycoforms”; being fucosylated core-*α*-(1,2−&−1,3), bisecting, in addition (bi-, tri- and tetra-) antennary glycans expression could be represented in all animals groups, as conceivable continuation of the homeostasis phenomenon except for some limitations in SCID (10 dpi). SCID mice had significantly high-mannose *N*-glycans (2.56 *µ*M) than BALB/c mice control (1.39 *µ*M) due to the effect of *mannosyltransferase* gene in *Toxoplasma* infection^13^. However, mice infected with acute *T. gondii* and further treated with 1-MT had abundant increasing of fucosylated, bisecting *N*-glycans and particularly *sial*-1, *sial*-2 *N*-glycans (9.42, 0.66, 25.86 and 221.77) compared to the BALB/c control (7.6, 0.23, 11.5 and 83.3), respectively. This may explain the therapeutic efficiency of 1-MT and it’s overcome to infection. The MT drug diminished *T. gondii-*induced anhedonia and despair; also it revealed the possible effects of this drug against psychological disorders (e.g., depression). Moreover, this drug is capable of penetrating the blood-brain barrier and inhibiting *T. gondii* growth^42^. Further 1-MT inhibits *Toxoplasma* growth *in vitro*; they may be a potential therapy for *T. gondii* of complicated depressive symptoms. Importantly, the major peaks of *N*-glycans in all experimental groups were significantly sialylated-terminals compared to BALB/c control. The more interesting point is that fucosylated (core-*α*-1, 6) *N*-glycan models were greatly expressed in parasitic infection than control, (Peak #s 3, 5; Figs. 4, 5, and 6 in experiments I, II, and III, respectively), (Table 1) and (Table 2, *P*<0.001, *P*<0.001, *P*<0.01, in experiment I, II, III, respectively). It was indicated the first step of the immune system to survive the infection^16^. Meanwhile, the presence of core-fucose at IgG *N*-glycan regulates antibody-dependent cellular toxicity and complement activity^42^. Therefore, adapting to *T. gondii* infection may happen through three possible pathways; i) in chronic *T. gondii* infection (40 dpi), a long-term stress negatively influences animal fitness through *T. gondii* bradyzoite cysts (brain cysts) which might result in a mild encephalitis; otherwise it related to major influences of the parasitic infection on the brain; ii) in *T. gondii* infection (10 dpi), a short-term stress revealed depressive-and sickness-like behaviors. In order to refresh the fitness and to activate the immune system, administration of 1-MT as an immune-stimulating drug was recommended; and iii) in SCID mice (10 dpi), its T- and B-cells of the adaptive immune system were probably damaged because of the genetic mutation. Infected SCID mice (10 dpi) may neither resist the infection nor affect their fitness and their homeostatic immune failure ended by death. Altogether is confirmed by peaks of MS spectra (Figs. 4, 5, and 6) and the evident level of anhedonia- and despair-like behaviors (Fig. 1A–C) in experiment I-III, respectively. However, the clear modification of Neu5Gc into Neu5Ac-terminal(s) indicated the successful resistance of mice to acute/chronic *T. gondii* infection at the time of incubation period with limited/great fitness^43^. Thus, the data revealed the biological significance of changing Neu5Ac-into Neu5Gc-terminal for the regulation course of immune responses. It might indicate the connections between IgG-glycoforms and the receptors/lectins binding/PRRs of Fcγ/FcRn on dendritic cells^36^.

### Creation of glyco-biomarker of toxoplasmosis

Understanding the importance of the sialylated IgG *N-*glycans in Fc-region and the immune monitoring pathway in animal/human is useful. These targeted *N*-glycans were bind to Fcγ-receptor to moderate the quaternary complicated compositions, also to enhance the thermodynamic stability of the Fc-region^44^. Therefore, sialylated Fc-*N-*glycans was initiated in human-IgG to deal with the health and/or psychological defects^45^. Moreover, the connection of sialylated IgG Fc-*N-*glycans and dendritic-cell-specific ICAM-3 grabbing non-integrin (DCSIGN in human/SIGN*-*R1 in mice) might use as stimulant factors for overcoming the inflammatory process and/or depression through identifying an endogenic factor to control the T_h_2 mechanical pathway to sustain the body homeostasis^46^. Furthermore, it was reported that lectin binding *sia*-*α2*-6Gal/GalNAc was regularly changed the glycan compositions in depressed-persons. In addition, the expression of *sialyltransferase* gene of *ST6GALNAC2* was reduced in total leukocytic accounts in depressed human model^47^, also *ST8SLA2* that encoded *α*-2,8- *sialyltransferase* 8B was detected to be accompanied with the high risk to neuropsychiatric disorders^48^. Altogether, it makes sialylated glycomics a promising novel biomarker of depression and/or anxiety. The latest results were in line with our results that terminated- Neu5Gc *N*-glycan was significantly increased in chronic/acute *T. gondii* infection and genetic immunodeficiency of mice sera compared to controls (Figs. 4, 5, and 6 in experiments I, II, and III). Similarly, it well-known that the ratio between total plasma *sialyltransferase* and cortisol was impacted by neural function of the hypothalamic-pituitary-adrenal axis in either depressed-or schizophrenic-patients^15^. Herein, the *N*-glycans terminated with *sial*-group covered more than 50% of the whole *N*-glycan structures in mice sera (see Table 3). Moreover, it could be used markers of parasitic influences in depressive- mice model due to the activity of *sialyltransferases*^49^. Sialylation has been interconnected to neuropsychiatric defects; for instance, prion^50^, schizophrenia^51^, Alzheimer’s, and Huntington’s diseases^24,52^. Recently, the reason for depression is predicting unusual neural paths in response to external stimuli (e.g., stressors), resulted from maladaptive genetic and cellular elevations^53^. It leads to impaired function of these paths, and the failure of homeostasis^54^, or atrophy in neurons of the central nervous system of the diseases in child- or in adult-hood ages^24^. It may indicate post-translational glycosylation resulting from the changes in the biosynthesis of the *N*-glycome profiles. However, serum *N*-glycoforms BALB/c mice in *T. gondii* (40 dpi), acute infection (10 dpi) treated with 1-MT and SCID mice (10 dpi) increased the sialylation of Neu5Gc compared to Neu5Ac-terminal. This phenomenon was represented in Peak #s 4~12. The extreme exhausting of Neu5Gc-terminal in Peak # 15 revealed the opposite influences of chronic *T. gondii* on almost mass expressions of glycans in mice sera (Tables 1, 2 and Figs. 4, 5, and 6 in experiments I, II, and III, respectively). Although the direct correlation to the clinical scoring of infected BALB/c or SCID mice (Table 5B, C, and D) was emphasized at Peak # 15, no scoring had been stated in a chronic *T. gondii* infection (40 dpi) that might be due to the adaptation of animal in a long-term infection (Table 5A). It was beyond our expectation that this adaptation may be caused by a long-term impairment of the homeostatic immune balance in BALB/c mice. It was considered one of physiological compensatory trials to cope with the *T. gondii* infection (10 dpi) without behavioral findings significances. However, it caused a short-term impairment of the homeostatic immune balance in the BALB/c mouse, in acute *T. gondii*, due to therapeutic interference (1-MT). It was notably characteristic candidates (Peak # s 14 and 15) which soundly correlated with the behavioral observations had extreme biological importance for the prognosis of depression. All these patterns typically demonstrated through the *N*-glycomics and animal behavioral data interaction (shown in Table 5 A, B, C, and D in experiments I, II, and III). So, our result agreed with the previous study^55^, who reported that the *N-*glycosylation profiles were clearly altered in diseased-persons from those healthy controls. All the above-mentioned factors are probably associated with the strong impact of sialylation on the neural function and depression in human/animal^56^.

### Neu5Gc/Neu5Ac ratio of toxoplasmosis

The changing of Neu5Gc into Neu5Ac-terminal is a master to indicate the regulatory mechanism of immunity in mouse and to refer whether the mouse is in a depressive state. Moreover, Neu5Ac/Neu5Gc ratio was altered significantly among the majority of *N-*glycoform profiles expressed in mice infected with chronic and/or acute *T. gondii* infection (Figs. 4, 5 in experiments I, II) and (Tables 1,2) compared to the controls. However, the reason how precisely Neu5Gc-converted into Neu5Ac-terminal is still indistinct and requires additional researches. The upregulation of serum Neu5Gc-terminal probably explained the long/short-term impairment (Peak #s 4~12), which launched during chronic/acute *T. gondii* infection in mouse. Furthermore, the depressive-like behavior was revealed through the novel expression threshold of the Neu5Gc-terminal, particularly in Peak # 15 of the infected mouse. Therefore, quantitative analysis of the expressed Neu5Gc-terminal may affluence early prognosis for depression degree in animal/human model by a direct reflection on their general immunity/health, behaviors, and performances. Herein, BALB/c mice had chronic *T. gondii* infection (40 dpi) or treated with 1-MT in acute *T. gondii* (10 dpi) can resist the infection by consuming nearly twice concentrations, 225 ± 10.7 *µ*M or 215 ± 4.7 *µ*M, respectively, *P*<0.001 each) of sialylated *N*-glycan (Neu5Gc-terminal, Peak # 15) than BALB/c control (108 ± 5.9 *µ*M), data shown in Tables 1, 2. However, SCID mice infected with *T. gondii* succumb after 10 dpi seemed to consume three times concentration (301 ± 17.3 *µ*M, *P*<0.001) of Neu5Gc-terminated *N*-glycan (Peak # 15) than in BALB/c-controls. By which, the importance of *sial*-terminals ratio is needed to regulate the body homeostasis of mice. Consequently, our findings (Table 6) clearly suggested that sensitive glyco-biomarker (Peak # 14), with 2 × Neu5Ac-terminals was sufficient proof of the excellent point of body fitness. For instance, Peak # 14 was represented as follows; chronic *T. gondii* infection (BALB/c mice can resist the chronic infection, (2.0 ± 0.2* µ*M), the potential role of 1-MT treatment in acute infection, (2.84 ± 0.2* µ*M), and the uninfected SCID mice, (4.51 ± 1.1* µ*M), (see Figs. 4, 5, and 6, for experiments I, II, and III, respectively, *P*<0.001 each). Then, there was a continuation of apparent normal homeostasis. The abundant expression level of Peak # 15 with 2 × Neu5Gc-terminals in mice was a typical biomarker of the compensatory mechanism of depressive-like behaviors. Thus, it came through the fact that further peak # s 16~19 identified in chronic/acute *T. gondii* infection; were suggested the sensitivity of host defense mechanism/the efficiency of the therapeutic agent against *T. gondii* infection (Fig. S1, S2 in Supplementary Information, for experiments I, II, respectively). However, no peaks detected afterword, suggesting a clinical death due to a failure of homeostatic immune balance as in SCID (10 dpi), (Fig. S3, in Supplementary Information, for experiment III). Our result agreed with our previous study reported and revealed a failure of homeostatic immune balance after exposure to chronic unpredictable mild stressors in BALB/c mice^18^. Thus, we have significantly discovered *N*-glycan expression in adaptive immunity due to the dynamic infection variations. In particular, chronic, acute *T. gondii* infection (Figs. 1A,B and 4, 5, for experiments I and II, respectively) and confirming the role of glycomics in innate immunity, for experiment-III. Meanwhile, the expression of multi-sialylation was ultimately linked to *T. gondii* infection which can absolutely affect the *sialyltransferase* and *sialidase* enzymatic activities. Moreover, it was realized that the biosynthetic pathway (Figs. 4, 5, and 6, for experiments I, II, and III, respectively) of the detected *N*-glycans have been released through intra-cellular concentrations of numerous sugar molecules, and the evidence of enzymatic activities.

### Limitation of the study

There was no possibility to detect the lectins with binding affinities for glycan structures and to quantify the amount of sialylated *N*-glycan biomarkers by 1,2-diamino-4,5-methylenedioxy-benzene-high-performance liquid chromatography (DMB-HPLC) analysis. However, this research is essential and acts as the first step to diagnose the influence of *T. gondii* infection in early stages and to confirm the impact of the antidepressants (1-MT) on the alteration of serum glycoproteins/glycans. Thus, we reveal that this work has great diagnostic criteria, particularly since the glycomics findings were in a correlation to the animal behavioral records. We succeeded to have the validity in the development of serum-based glyco-biomarkers in *T. gondii* infection during immune-competent and immune-compromised states. In addition, we illustrated the serum glycan’s expression for the therapeutic efficiency of 1-MT against *T. gondii* infection *in vivo*.

## Conclusion

Glycomics profiling is a novel technique to highlight the behavioral patterns of BALB/c mice under the condition of *T. gondii* infection. Eighteen major *N*-glycans were detected after displaying the glycoblotting along with the MALDI-TOF/MS analysis in mice sera. The alteration of sialylated *N*-glycan expressions through the biosynthetic pathway is important to detect the levels of immunity thresholds along with the adaptation of animal’s behavioral patterns to parasitic infection. Neither depressive-nor sickness-like behaviors were observed in chronically infected immunocompetent mice with *T. gondii*, while sialylated *N*-glycans were more expressed than in controls. However, depressive-and sickness-like behaviors were significantly developed after acute *T. gondii* infection in BALB/c mice. It was related to the high expression levels of immunostimulant *N*-glycan terminated with Neu5Ac (Peak # 14) residues and immunosuppressant *N*-glycan terminated with Neu5Gc residues (Peak # 15), accordingly *in vivo*. The successful antidepressant, 1-MT treatment, may downregulate immune system activation against *T. gondii* in immune-competent mice. Moreover, sickness but not depressive-like behaviors abundantly developed in infected immune-competent mice due to the high expression level of Peak # 15. The modification of Neu5Gc-to Neu5Ac-residue(s) acts as the master key that adjusts the level of homeostasis either by host immune defense or by a potential therapy like 1-MT. Therefore, Neu5Ac/Neu5Gc ratio is probably directed our thought to create a novel glyco-biomarker of depression. Our finding probably has promising implications for researchers in making an early prognosis to depression.

## Material and Methods

### Animals

This study was conducted, from July 2018 to March 2019, at the animal laboratory facility, Faculty of Medicine, Sohag University, 82524. All animals were treated humanely. Moreover, the standards and regulations of animal care have been applied carefully. Our study was approved by the Committee Members of Animal Ethics and Welfare, Faculty of Medicine, Sohag University, protocol number (161019-04-2018), and therefore all methods were performed in accordance with the relevant guidelines and approval of that committee. After the animals were anesthetized, through isoflurane, all injections have been made in order to keep the animal welfare and minimize the pain as possible. This study utilized seventy-two (n = 72) female BALB/c mice (7–9 weeks old), non-pregnant, ranged weight 24 ± 1.3 g. Mice were obtained from The Experimental Animal House, Theodor-Bilharz’s Research Center, Egypt. Moreover, the animals were kept under ambient environmental in Animal Laboratory Facility at Medicine Faculty. Mice were recommended to be reared for a week in identical environmental condition (12-h light: 12-h dark; turning the light from 7 AM to 7 PM) with providing an *ad-libitum* source to food and water at 09:00 AM, prior starting all behavioral tests.

### Parasite and infection with *T. gondii*

*T. gondii* type-II (PLK) strain tachyzoites were kept in cultured African green monkey kidney epithelial (Vero) cells. Further, a perfect purification of this stain was performed based on the previous report^57^. Then, intraperitoneal infection with 10^3^ tachyzoites kept in 0.2 mL sterile phosphate-buffered saline (PBS) per each experimental mouse. Furthermore, mice were decapitated, and 0.5–1.0 ml of the blood was collected perfectly. We kept the clotted blood at 4 °C for 16 h and proper centrifugation was done at 5000 g for up to 10 min. Finally, mice sera were transferred into new tubes in order to keep them at −80 °C ready to be used on the day of analysis.

### Treatments and experimental groups

All mice were regularly handled before the treatments. As shown in Table S6 in Supplementary Information, the BALB/c mice groups (n = 72 in total, n = 8/group) used in three experiments, as follows: (i) experiment-I [group-1: control BALB/c mice that were daily exposed to gentle management for the equivalent period and injected with PBS. Group-2: BALB/c mice were infected with *T. gondii*, 40 dpi, was the endpoint)]; (ii) experiment-II [group-1: control (vehicle-injected mice), group-2: BALB/c mice were exposed to *T. gondii* infection, group-3: BALB/c mice were administrated with 1-MT, and group-4: BALB/c mice were infected with *T. gondii* followed by treatment with 1-MT] - the endpoint was at 10 dpi; and (iii) experiment-III [group-1: control BALB/c mice (PBS-injected mice), group-2: BALB/c mice that were infected with acute *T. gondii*, (10 dpi), group-3:SCID mice, and group-4: SCID mice were infected with acute *T. gondii* (10 dpi). Noticeably, at 4 dpi, groups of *T. gondii*-infected and PBS-injected mice were subcutaneously treated with indoleamine dioxygenase inhibitor (IDO) 1-_DL_-MT (50 mg/kg) or its vehicle was given once a day and continued up to 4 days^58^. The 1-_DL_-MT was dissolved in 0.1 N hydrochloric acid (HCl), neutralized with 0.1 M sodium hydroxide (NaOH), buffered with 2× PBS, and then perfectly purified through 0.2 *µ*m of syringe filter before use.

### Behavioral measurements

#### Sucrose preference test

a reduction in sucrose consumption putatively indicates anhedonic-behavior^59^. The test was performed based on a reward test protocol^6,59^. Mice were adapted with a couple of bottles of water for a week, followed by 1% sucrose and water (one bottle each). Furthermore, the total amount of fluid intake was estimated daily. Moreover, the ability of mouse for sucrose preference was detected as a sucrose intake % to the sum of water and sucrose intakes.

#### Forced swim test (FST)

FST was done based on the previous report^3^. Briefly, FST was performed using a normal light program up to 6 min. Additionally, immobility score has been detected as the time in which mice remain floating motionless during 4 min where the first 2 min of the test were excluded for adaptation. Mice were separately transferred into the water-filled FST cylinder. The FST cylinder is a Pyric glass cylinder; 12-cm diameter, filled to 25-cm water depth and kept at 10 °C. After the time of the test, mice were dried using a paper towel and rapidly returned to their home-cages. The analysis was accomplished blindly by a well-trained observer.

#### Assessment of locomotor activity

Mice’s locomotion was assessed in a clean cage matching to their home-cage. Test cages kept within the same housing condition, but without bedding, as described previously^60^. In a cage, every line crossing and rearing of mouse was quantified over 12 virtual identical quadrants through 180 s duration time. The general activity is measured as line crossings and rearing in a cage.

#### Clinical score

Ethograms were displayed based on the presence of clinical signs of mice during *T. gondii* inside their home-cages, according to the previous report^6^. Values varied from 0 to 10, no signs, all sigs, respectively. In brief, the observed signs included posture such as (ataxia, warmth-seeking behavior, reluctant movement, hunching, and lying on the belly), eye condition (ptosis, sunken eyes), pilo-erection, and deficient evacuation and touch reflexes. Each sign worth 1 point, and there were a total of 10 signs measured.

### Glycoblotting-based serum glycomics

Glycoblotting was carried out using mice sera (n= 288) in order to quantify *N*-glycomes. Whole *N*-glycomics were analyzed as described before with minor modulations^18,20,24,61^. Mouse sera glycoproteins were achieved in order to purify the entire *N*-glycans using PNGase F enzyme. Further, selective capturing of glycans was performed through glycoblotting-technique using the bead of BlotGlyco^®^H. The esterified methyl-group of sialic acid residue(s) and transamination reaction was done for tagging *N*-glycans using BOA on the glyco-beads. As a final point, MALDI-TOF/MS analysis was applied to BOA-labeled *N*-glycans (illustrated in Fig. 3). The glycomic procedures are as follows: A) *N*-glycan release; B) chemoselective ligation; C) washing; D) on-bead methylation of sialic acid(s); E) *trans*-amination reaction; and F) MALDI-TOF/MS analysis. The detailed information on the chemicals and glycomic strategy is mentioned in Supplementary Information I, II.

### Statistical analysis

Assessments of *N-*glycans in mice sera were expressed as micromolar, M ± SD. The statistical alterations among groups were utilized using Student’s *t*-test and one/two-way-ANOVA repeated measures. A comparison of variables was done using non-parametric tests. The concentration (*μ*M) of *N*-glycans of multiple molecular masses (*m/z*) was represented as the dependent variable. The group whether controls or experimental groups was the fixed factor. Moreover, parameters such as sucrose preference in %, immobility duration/ sec, locomotor activity/line crossing/ 3 min and clinical scoring were represented as covariates to show the behavioral changes after exposure to the *T. gondii*. We used parametric testing because, in general, our results were just normally distributed. The mean effects showed significance when *P*-value was <0.05. In addition, we used *GraphPad Prism* 5 to show Pearson’s correlation coefficients of behavioral patterns of individual samples in a scattered dot plot. Altogether, it was to examine the appropriate interactions among the groups.

## Supplementary information

Supporting information

## Data Availability

The corresponding author(s) have all data represented in this manuscript for any request.
