# Supplementary material for: Sialylated N-glycan profile during acute and chronic infections with Toxoplasma gondii in mice
Source: Sci Rep. 2020 Mar 2;10:3809. doi: 10.1038/s41598-020-60681-4 (PMC7052212; doi:10.1038/s41598-020-60681-4)
Supplement: Supplementary file 1 — Supporting information [file 41598_2020_60681_MOESM1_ESM.docx]

**Supporting Data:**

**Sialylated *N*-glycan profile during acute and chronic infections with *Toxoplasma gondii* in mice**

*Ibrahim Farag Rehan****^1,*^****,* *Motamed Elsayed Mahmoud****^2,*^****,* *Doaa Salman****^3^****,* *Asmaa Elnagar****^4^****, Saleh Salman****^5,6^****, Mohammed Youssef****^7^*** *,* *Amer Rageb Abdel Aziz****^8^*** *, Eman Kamal Bazh^9^, and Abd El-Latif Hesham****^10,*^***

**Author information**

**Affiliations**

**[1]** Department of Husbandry and Development of Animal Wealth, Faculty of Veterinary Medicine, Menofia University, Shebin Alkom, Menofia, 32511, Egypt.

**[2]** Department of Animal Behavior and Husbandry (management, genetics, and breeding), Faculty of Veterinary Medicine, Sohag University, Sohag, 82524, Egypt.

**[3]** Department of Animal Medicine, Faculty of Veterinary Medicine, Sohag University, Sohag, 82524, Egypt.

**[4]** Department of Biochemistry, Faculty of Veterinary Medicine, South Valley University, Qena, 83523, Egypt. **[5]** Department of Animal Sciences, Colorado State University, Fort Collins, 80523, Colorado, USA.

**[6]** Department of Animal Production, Faculty of Agriculture, Assiut University, 71111, Egypt.

**[7]** Department of Animal Physiology, Faculty of Veterinary Medicine, South Valley University, Qena, 83523, Egypt.

**[8]** Department of Parasitology, Faculty of Veterinary Medicine, Sohag University, Sohag, 82524, Egypt.

**[9]** Department of Parasitology, Faculty of Veterinary Medicine, Menofia University, Shebin Alkom, Menofia, 32511, Egypt.

**[10]** Department of Genetics, Faculty of Agriculture, Beni Suef University, Beni Suef 62511, Egypt.

**Corresponding authors**

Correspondence to Ibrahim Farag Rehan, Motamed Elsayed Mahmoud and Abd El-Latif Hesham.

*E-mail address: [ibrahim.rehan@vet.menofia.edu.eg](mailto:ibrahim.rehan@vet.svu.edu.eg) (Ibrahim Farag Rehan), [motamed71111@gmail.com](mailto:ibrahim.rehan@vet.svu.edu.eg) (Motamed Elsayed Mahmoud) and hesham_egypt5@aun.edu.eg (Abd El-Latif Hesham).

**The detailed information of the strategy is as the following:**

**I- Chemicals and equipment used**

Ammonium bicarbonate 99% (ABC), cultured African green monkey kidney epithelial (Vero) cells, 1-propanesulfonic acid, 2-hydroxyl-3-myristamido (PHM), 1,2-diamino-4,5-methylenedioxy-benzene (DMB), 3-Methyl-1-*p*-tolyltriazene (MTT), disialyloctasaccharide and *O*-benzylhydroxylamine hydrochloride (BOA) were purchased [Tokyo Chemical Industry Co., Ltd., Tokyo, Japan]. BlotGlyco^®^H beads were obtained from Sumitomo Bakelite Co., Ltd. (Tokyo, Japan). Phosphate buffered saline (PBS), peptide *N*-glycosidase F (PNGase F) and *T. gondii* strain tachyzoites were obtained [New England Biolabs^R^ Co., Inc., Ipswich, USA]. SweetBlot^TM^ (included automated glycan processing and the incubation machine) [Systems Instruments Co., Inc., Hachioji, Japan]. Trypsin, 1-methyl tryptophan (1-MT), dithiothreitol (DTT), iodoacetamide (IAA), isoflurane and indoleamine dioxygenase inhibitor (IDO) were purchased from [Sigma-Aldrich, Co., Inc. (St. Louis, MO, USA]. PLK strain of *T. gondii* was provided from National Research Center for Protozoan Diseases, Obihiro, Hokkaido, Japan. Mass measurements were generated using MALDI-TOF/MS machine (Ultraflex III and FlexControl & FlexAnalysis 3.0 version Software), [Bruker Daltonics, Germany] and SPSS-software was obtained [IBM Co., Inc. Armonk, NY, USA]. MultiScreenSolvinert^R^ filter plates were purchased [Millipore Co., Inc. (Tokyo, Japan]. Otherwise other chemical reagents and solvents were prepared using [Wako Pure Chemicals Co., Ltd., Tokyo, Japan].

**II- Glycoblotting-based serum glycomics**

***(A) N-glycan release*:** fifty *µ*L of [0.33 M ABC (2.52 mL), 120 mM DTT (0.84 mL), 0.4% PHM in 10 mMABC (1.26 mL), and further addition of 3.78 mL Milli-Q water] was prepared, as a premix composition. This mixture was added to 10 *µ*L serum of each mouse. 40 *µ*M disialyloctasaccharides (internal standard, I.S), with a chemical composition [(Hex)_2_ (HexNAc)_2_ (NeuAc)_2_ + (Man)_3_ (GlcNAc)_1_], was added carefully and then mixed in each well. The mixture was then kept at 60^o^C for 30 min. The alkylation process was conducted by addition of 20 *µ*L of 123 mM IAA and then incubated in the darkness place for 1 hr. Then, the mixture was enzymatically interacted by addition of 10 *µ*L of 40 U/*µ*L trypsin dissolved in 1 mM HCl at 37°C for 3 hrs. Heat inactivation was done at 90°C for 10 min then stopping the reaction by remaining the mixture at room temperature. *N*-glycans were generated from trypsin-digested samples through incubation with 2 U of PNGase F at 37°C for 6 hrs. Then, 60 *µ*L of a digested mixture containing *N*-glycans was directed to glycoblotting technique. The glycoblotting is performed through the following steps (B-E):

***(B) Chemoselective ligation*:** twenty hundred and fifty *µ*L of BlotGlyco^®^H beads; dissolved in a 10 mg/mL suspension of Milli-Q water; was transferred into each well of a MultiScreen-Solvinert filter plate with a vacuuming. The digested mixture of released *N*-glycan of 20 *µ*L mouse serum 0 *µ*L was mixed regularly with the beads in each well. Consequently, 80 *μ*L of 2% acetic acid AcOH in acetonitrile (CH_3_CN) was added carefully. In order to selectively capture the *N*-glycans onto the beads through stable hydrazine-bonds, the plate was kept at 80°C for 45 min till complete dryness in a thermostat.

***(C) Washing***: the plate is recommended to wash twice with each 200 *μ*L of 2 M guanidine-HCl after dissolving in ABC, Milli-Q water as well as 1% triethylamine after dissolving in MeOH. Then, capping of the functional acetyl group of unreactive hydrazide was performed by addition of 10% acetic anhydride after dissolving in MeOH and then incubated at 25°C for 30 min. Moreover; the remaining of acetic anhydride was vacuumed.

**(D) *On-bead methylation of sialic acid(s)*:** the beads were washed twice with 200 *μ*L of 10 mM HCl, MeOH and followed by dioxane. Then, freshly 150 mM MTT in dioxane was added to inhibit sialic acid(s) dissociation under whether the acidity-condition or direct ionization by the MALDI-TOF/MS. The plate was incubated at 60^o^C for 90 min. Therefore, every well was double-washed using 200 *μ*L of dioxane, Milli-Q water & methanol, Milli-Q water.

**(E) *Trans-amination reaction*:** labeled of the glycan blotted on the beads was done by *trans*-aminization reaction using 20 *μ*L of 50 mM BOA, and also 180 *μ*L of 2% AcOH dissolved in CH_3_CN with an incubation at 80°C for 45 min. Finally, the elution of BOA-tagged *N*-glycans were performed carefully with 100 *μ*L of Milli-Q water and then directly subjected to SweetBlot^TM^.

The analysis of BOA-tagged *N*-glycans were performed after the spotting process on MTP 384 targeted-plate and then vacuumed to having it in a crystal form with the same volume of matrix solution. The matrix used was in a liquid form (100 mM *α*-cyano-4-hydroxycinnamic acid diethylammonium salt). It has been dissolved in a buffer solution (MeOH: Milli-Q water: DMSO: 10 mMNaOH= 50: 39: 10: 1). The data of MALDI-TOF/MS was then monitored on the Ultraflex III using AutoXecute flexControl Software based on the general protocol. Therefore, automatic assignments were done prior to operating FlexControl software to have quantitatively reproducible spectra in all subjects. The spectral conditions was adjusted to be displayed using the reflector, positive-ion mode generated by Smartbeam (pulsed UV solid laser, λ_ex_= 355 nm, 50 Hz), the acceleration voltage (25 kV), the reflector voltage (26.3 kV), pulsed ion extraction (160 ns) and summing up to 1000 shots per one spot. The total mass-spectra were then analyzed using FlexAnalysis (version 3.0 Software). Adjusting an internal standard (40 *μ*M disialyloctasaccharide) was highly important to acquire the standard intensities of the monoisotopic peaks of each quadruplicated spectra. Further, the estimation of the concentration of those peaks was done according to the standard curve of the samples of human sera with known concentrations and then averaged. In addition, the difference between calibrated and observed masses was electronically defaulted to be less than 0.5 Da; however, ppm error was adjusted to be below 500.

***(F) MALDI-TOF/MS:*** detected *N*-glycans were nominated according to special criteria of their quantitative reproducibility using MALDI-TOF/MS in order to categorize the development of depressive-like behaviors in mice sera. For further detection of the potential structures of *N-*glycans, GlycoMod web-based tool (cited: http://web.expassy.org/glycomod/) was utilized with an input of experimental molecular masses which were commonly sourced either GlycosuiteDB, or non-reported compositions in Consortium for Functional Glycomics, (CFG), (cited: http://www.functionalglycomics.org). *N*-glycan structural characteristics were principally chosen according the following priority from the *Mus Musculus* species sera, cells or tissues origins then *Rattus norvegicus* and human sera origins where considered sequentially.

**Table S1** Comparison between observed m/z and theoretical *m/z* values in in BALB/c and SCID mice serum

| **Peak**  **#** | **Observed *m/z*** | **Theoretical *m/z* with the sialic acid(s)/reducing end modifications** | **Mass differences** |
| --- | --- | --- | --- |
| **1** | 1362.48109 | 1234.43347 | 105+23 |
| **2** | 1524.53392 | 1396.48630 | 105+23 |
| **3** | 1590.59210 | 1462.54448 | 105+23 |
| **4** | 1724.61363 | 1582.55036 | 105+23+14 |
| **5** | 1752.64493 | 1624.59731 | 105+23 |
| **6** | 1870.67154 | 1728.60827 | 105+23+14 |
| **7** | 1886.66646 | 1744.60319 | 105+23+14 |
| **8** | 1898.70284 | 1770.65522 | 105+23 |
| **9** | 1927.69301 | 1785.62974 | 105+23+14 |
| **10** | 2029.72470 | 1873.64578 | 105+23+28 |
| **11** | 2048.71929 | 1906.65602 | 105+23+14 |
| **12** | 2089.74584 | 1947.68257 | 105+23+14 |
| **13** | 2175.78261 | 2019.70369 | 105+23+28 |
| **14** | 2378.86199 | 2222.78307 | 105+23+28 |
| **15** | 2410.85183 | 2254.77291 | 105+23+28 |
| **16** | 2425.88788 | 2297.84026 | 105+23 |
| **17** | 2746.99387 | 2604.9306 | 105+23+14 |
| **18** | 3112.12608 | 2970.06281 | 105+23+14 |
| **19** | 3140.15738 | 3012.10976 | 105+23 |
| The mass differences between the observed *m/z* and theoretical *m/z* were due to the “R” group in an oxyamine used to cleavage the glycans from beeds and methylation of sialic acid residues. *N*-glycans were labeled with BOA for *trans-*amination (105 *m/z*), formation of Na adduct (23 *m/z*) by laser condition during shooting to demonstrate MS spectra, and esterification of sialic acid per one or two methyl group (14 *m/z*, 28 *m/z*). After adding these reactive *m/z* values to the theoretical *m/z* either with or without sialylation, we get the typical observed *m/z* values. | | | |

**Table S2** Pearson’s correlation coefficients of sucrose preference%, immobility duration (sec), line crossing (every 3 min), and particular peaks levels of serum *N*-glycans in *T. gondii* chronically infected mice.

| ***N*-glycans**  **Peak #** | **Sucrose preference (r)** | **Immobility duration (r)** | **Line crossing**  **(r)** |
| --- | --- | --- | --- |
| 1 | 0.472* | 0.016 | -0.471* |
| 2 | 0.492* | 0.034 | -0.451* |
| 3 | -0.032 | 0.684** | 0.652** |
| 4 | 0.606** | -0.284 | -0.726*** |
| 5 | -0.038 | 0.946*** | 0.699** |
| 6 | 0.346* | -0.889*** | -0.726*** |
| 7 | 0.206 | -0.824*** | -0.584** |
| 8 | 0.600** | 0.642** | -0.077 |
| 9 | -0.256 | -0.605** | 0.0144 |
| 10 | 0.656** | -0.652** | -0.931*** |
| 11 | 0.206 | -0.226 | -0.340* |
| 12 | 0.298 | -0.134 | -0.095 |
| 16 | -0.624** | -0.629** | 0.101 |
| 17 | -0.058 | 0.024 | -0.005 |
| 18 | 0.376* | 0.368* | -0.037 |
| 19 | -0.401* | 0.729*** | 0.904*** |

Each value represent Pearson’s correlation coefficients *** [r] = 0.7, strong correlation, **, [r] = 0.5–0.7, moderately to strong correlation, and * [r] = 0.3–0.5, weak to moderate correlation.

**Table S3** Pearson’s correlation coefficients of sucrose preference%, immobility duration (sec), line crossing (every 3 min), and particular peaks levels of serum *N*-glycans in 10 day *T. gondii* infected mice

| ***N*-glycans**  **Peak #** | **Sucrose preference (r)** | **Immobility duration (r)** | **Line crossing (r)** | **Clinical Score (r)** |
| --- | --- | --- | --- | --- |
| 1 | 0.078 | 0.954*** | -0.001 | 0.377* |
| 2 | 0.168 | 0.961*** | -0.003 | 0.058 |
| 3 | 0.243 | 0.991*** | 0.069 | 0.301* |
| 4 | 0.340* | 0.989*** | 0.220 | 0.373* |
| 5 | 0.339* | 0.984*** | 0.053 | 0.344* |
| 6 | -0.184 | 0.214 | -0.903*** | -0.137 |
| 7 | 0.289 | 0.728*** | -0.451* | -0.157 |
| 8 | None | None | None | None |
| 9 | 0.223 | 0.842*** | -0.324* | -0.055 |
| 10 | 0.437* | 0.718*** | -0.387* | 0.460* |
| 11 | 0.460* | 0.898*** | -0.089 | -0.080 |
| 12 | 0.452* | 0.752** | 0.169 | 0.761*** |
| 16 | 0.555** | 0.935*** | 0.420* | 0.256 |
| 17 | 0.492* | 0.565** | 0.004 | 0.818*** |
| 18 | 0.414* | 0.778*** | -0.204 | 0.611** |
| 19 | 0.689** | 0.721*** | 0.066 | 0.583** |

Each value represents Pearson’s correlation coefficients (Pearson’s r): *** [r] = 0.7, strong correlation, **, [r] = 0.5–0.7, moderately to strong correlation, and * [r] = 0.3–0.5, weak to moderate correlation.

**Table S4** Pearson’s correlation coefficients of sucrose preference%, immobility duration (sec), line crossing (every 3 min), and particular peaks levels of serum *N*-glycans in 1-MT treated and *T. gondii* infected mice

| ***N*-glycans**  **Peak #** | **Sucrose preference (r)** | **Immobility duration (r)** | **Line crossing (r)** | **Clinical Score (r)** |
| --- | --- | --- | --- | --- |
| 1 | -0.598** | 0.717*** | 0.720*** | -0.320* |
| 2 | -0.324* | 0.399* | 0.736*** | -0.175 |
| 3 | -0.767*** | 0.837*** | 0.666** | -0.224 |
| 4 | -0.058 | 0.336* | 0.697** | -0.383* |
| 5 | -0.898*** | 0.707*** | 0.278 | 0.276 |
| 6 | -0.549** | 0.466* | 0.371* | 0.002 |
| 7 | -0.301* | 0.463* | 0.652** | -0.206 |
| 8 | -0.321* | 0.575** | 0.763*** | -0.383* |
| 9 | -0.353* | 0.633** | 0.848*** | -0.585** |
| 10 | 0.383* | -0.524** | -0.228 | 0.408* |
| 11 | 0.044 | -0.222 | -0.409* | 0.444* |
| 12 | 0.245 | -0.143 | -0.424* | -0.105 |
| 16 | 0.758*** | -0.575** | -0.172 | -0.165 |
| 17 | 0.585** | -0.432* | -0.199 | -0.111 |
| 18 | 0.158 | -0.330* | -0.667** | 0.404* |
| 19 | 0.141 | -0.382* | -0.656** | 0.539** |

Each value represent Pearson’s correlation coefficients *** [r] = 0.7, strong correlation, **, [r] = 0.5–0.7, moderately to strong correlation, and * [r] = 0.3–0.5, weak to moderate correlation.

**Table S5** Pearson’s correlation coefficients of sucrose preference%, immobility duration (sec), line crossing (every 3 min), and particular peaks levels of serum *N*-glycans in *T. gondii* infected SCID mice

| ***N*-glycans**  **Peak #** | **Sucrose preference (r)** | **Immobility duration (r)** | **Line crossing**  **(r)** | **Clinical Score (r)** |
| --- | --- | --- | --- | --- |
| 1 | 0.597** | 0.061 | 0.242 | -0.867*** |
| 2 | 0.174 | -0.393* | -0.219 | -0.551** |
| 3 |  |  |  |  |
| 4 | 0.338* | -0.232 | -0.051 | -0.685** |
| 5 |  |  |  |  |
| 6 | -0.008 | 0.541** | 0.379* | 0.404* |
| 7 | -0.966*** | -0.950*** | -0.991*** | 0.785*** |
| 8 | -0.993*** | -0.894*** | -0.961*** | 0.866*** |
| 9 | 0.909*** | 0.532** | 0.677** | -0.999*** |
| 10 | -0.729*** | -0.236 | -0.409* | 0.941*** |
| 11 | -0.651** | -0.960*** | -0.893*** | 0.295 |
| 12 | -0.087 | 0.472* | 0.304* | 0.476* |
| 16 | -0.763*** | -0.285 | -0.454* | 0.957*** |
| 17 |  |  |  |  |
| 18 |  |  |  |  |
| 19 | -0.970*** | -0.678** | -0.801*** | 0.987*** |

Each value represent Pearson’s correlation coefficients *** [r] = 0.7, strong correlation, **, [r] = 0.5–0.7, moderately to strong correlation, and * [r] = 0.3–0.5, weak to moderate correlation.

**Table S6** Design of experimental animal grouping

| **No.** | | **Grouping (n=72 in total, n=8/group)** | | | |
| --- | --- | --- | --- | --- | --- |
| **Exp.1** | CNT-BALB/c | | Chronic *T. gondii* |  |  |
| **Exp.2** | CNT-BALB/c | | Acute *T. gondii* | 1-MT | Acute *T. gondii* + 1-MT |
| **Exp.3** | CNT-BALB/c | | SCID | SCID + Acute *T. gondii* |  |
| Exp.1 [BALB/c-control, and BALB/c- exposed to chronic *T. gondii*], Exp.2 [BALB/c-control, BALB/c- exposed to acute *T. gondii,* 1-MT, and BALB/c- treated with 1-MT after exposure to acute *T. gondii*], Exp.3 [BALB/c-control, BALB/c- exposed to acute *T. gondii,* SCID-control, and SCID-exposed to acute *T. gondii*]. 1-MT: 1-methyl tryptophan; Exp.: experiment; and SCID: severe combined immune deficiency. | | | | | |


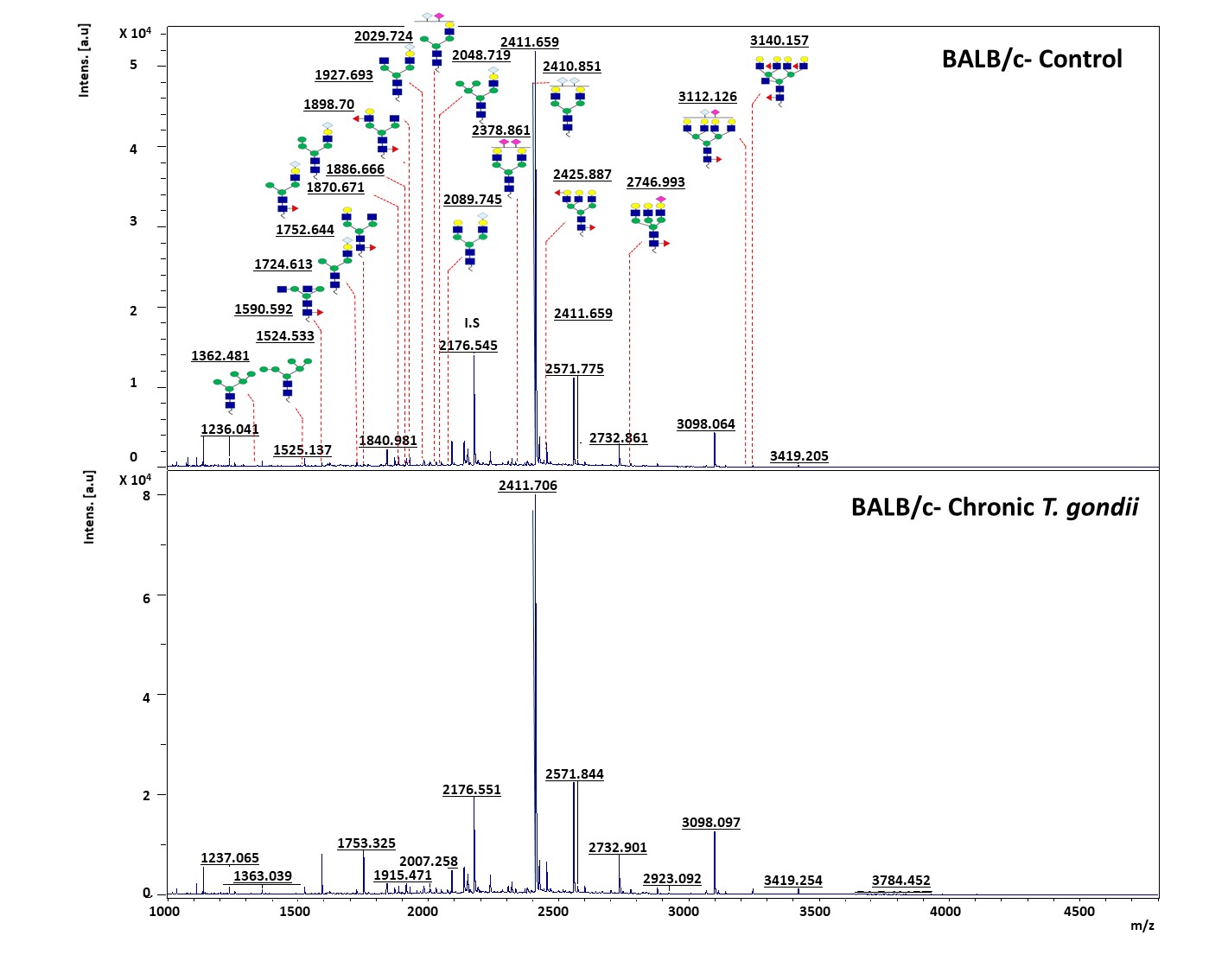
**Figure S1** Representative MALDI-TOF/MS spectra with typical serum *N*-glycan peaks and profiles in experiment-I. Experiment-I consisted of [BALB/c-control, and BALB/c- exposed to chronic *T. gondii* “40 dpi”], Peak # 13 identified at *m/z* 2176 which means the internal standard (IS) spiked (normalized as 40 *μ*M). MALDI-TOF/MS spectra are distinct in both groups of mouse serum.

**
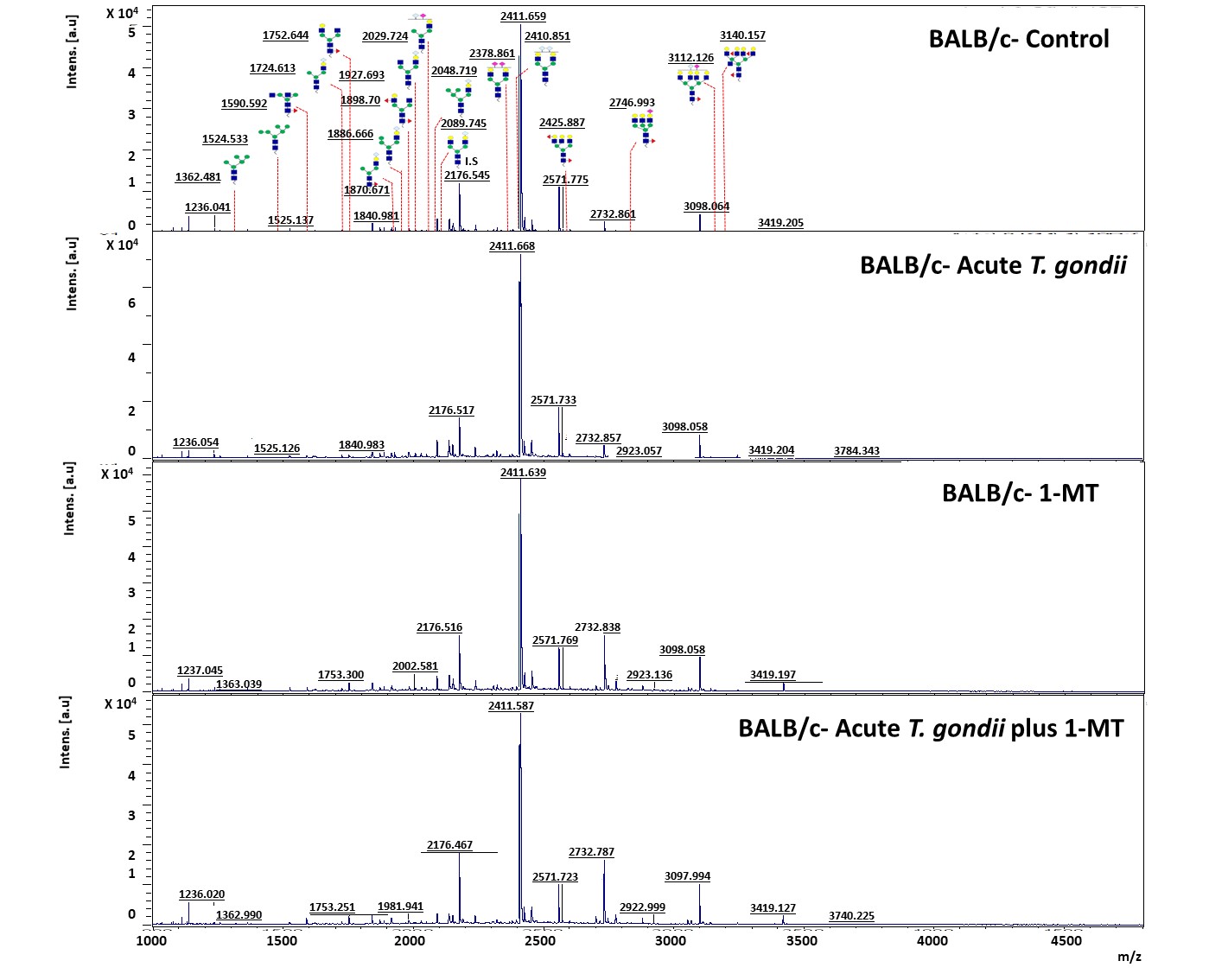
**

**Figure S2** Representative MALDI-TOF/MS spectra with typical serum *N*-glycan peaks and profiles in experiment-II. Experiment-II consisted of four groups, [BALB/c-control, BALB/c- exposed to acute *T. gondii* “10 dpi”, 1-MT, and BALB/c- treated with 1-MT after exposure to acute *T. gondii*]. Peak # 13 detected at *m/z* 2176 which means the internal standard (IS) spiked (normalized as 40 *μ*M). MALDI-TOF/MS spectra are distinct among groups of mouse serum. 1-MT: 1-methyl tryptophan.

**
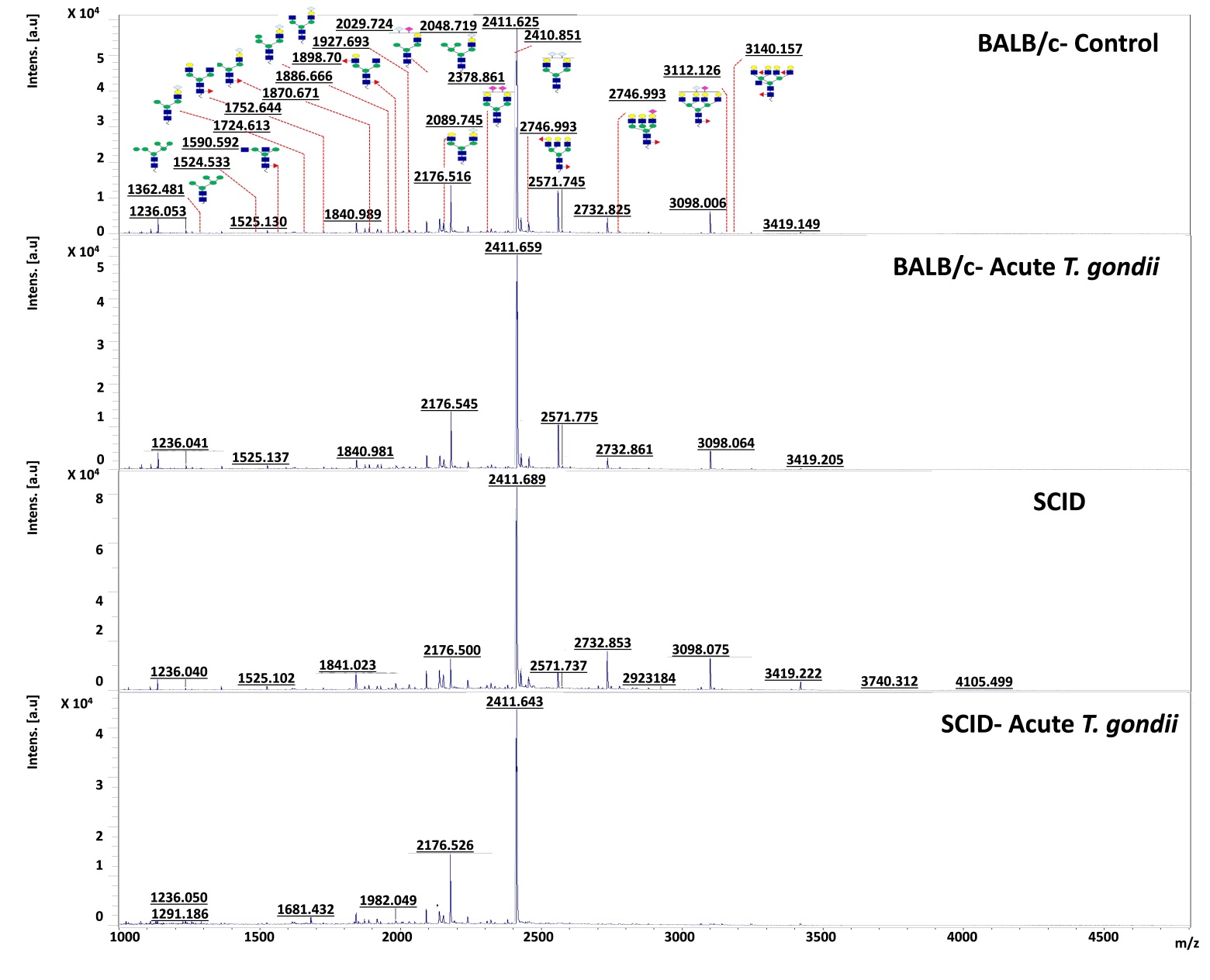
**

**Figure S3** Representative MALDI-TOF/MS spectra with typical serum *N*-glycan peaks and profiles in experiment-III. Experiment-III consisted of four groups, [BALB/c-control, BALB/c- exposed to acute *T. gondii* “10 dpi”, SCID-control, and SCID-exposed to acute *T. gondii* “10 dpi”]. Peak # 13 detected at *m/z* 2176 which means the internal standard (IS) spiked (normalized as 40 *μ*M). MALDI-TOF/MS spectra are distinct among groups of mouse serum. SCID, severe combined immunodeficiency.

**Control #1**

**Control #2**

**Control #3**

**Control #4**

**Figure S4** Representative MALDI-TOF/MS spectra (# s 1, 2, 3 and 4) showing typical *N*-glycan profiles of serum glycoproteins in experiment-I, II, III, in Gr. 1, group 1, control BALB/c mice.

**Chronic *T. gondii* #1**

**Chronic *T. gondii* #2**

**Chronic *T. gondii* #3**

**Chronic *T. gondii* #4**

**Chronic *T. gondii* #5**

**Figure S5** Representative MALDI-TOF/MS spectra (# s 1, 2, 3, 4 and 5) showing typical *N*-glycan profiles of serum glycoproteins in experiment-I, in Gr.2, group 2, BALB/c mice chronically infected with *T. gondii*, 40 days, PLK (strain forming brain cyst).

**Acute *T. gondii #1***

**Acute *T. gondii #2***

**Acute *T. gondii #3***

**Acute *T. gondii #4***

**Acute *T. gondii #5***

**Figure S6** Representative MALDI-TOF/MS spectra (# s 1, 2, 3, 4 and 5) showing typical *N*-glycan profiles of serum glycoproteins in experiment-II, III, Gr.2, group 2, BALB/c mice infected with acute *T. gondii*.

**1-MT #1**

**1-MT #2**

**1-MT #3**

**1-MT #4**

**1-MT #5**

**Figure S7** Representative MALDI-TOF/MS spectra (# s 1, 2, 3, 4 and 5) showing typical *N*-glycan profiles of serum glycoproteins in experiment-II, in Gr.3, , group 3, BALB/c mice treated with 1-MT (1-methyl tryptophan).

**Acute *T. gondii +*1-MT #1**

**Acute *T. gondii +*1-MT #2**

**Acute *T. gondii +*1-MT #3**

**Acute *T. gondii +*1-MT #4**

**Acute *T. gondii +*1-MT #5**

**Figure S8** Representative MALDI-TOF/MS spectra (# s 1, 2, 3, 4 and 5) showing typical *N*-glycan profiles of serum glycoproteins in experiment-II, Gr.4, , group 4, BALB/c mice infected of *T. gondii* and then treated with MT. 1-MT, 1-methyl tryptophan.

**Acute *T. gondii +*1-MT #2**

**SCID #1**

**SCID #2**

**SCID #3**

**Figure S9** Representative MALDI-TOF/MS spectra (# s 1, 2 and 3) showing typical *N*-glycan profiles of serum glycoproteins in experiment-III, in Gr.3, group 3, of SCID mice as control. SCID, severe combined immunodeficiency.

**SCID Acute *T. gondii* #1**

**SCID- Acute *T. gondii* #2**

**SCID- Acute *T. gondii* #3**

**Figure S10** Representative MALDI-TOF/MS spectra (# s 1, 2 and 3) showing typical *N*-glycan profiles of serum glycoproteins in experiment-III, in Gr.4, group 4, SCID mice infected with *T. gondii*. SCID, severe combined immunodeficiency.
